# 1,6- and 1,7-Regioisomers of Highly Soluble Amino-Substituted Perylene Tetracarboxylic Dianhydrides: Synthesis, Optical and Electrochemical Properties

**DOI:** 10.3390/ma8084943

**Published:** 2015-08-03

**Authors:** Kew-Yu Chen, Che-Wei Chang, Hsing-Yang Tsai

**Affiliations:** Department of Chemical Engineering, Feng Chia University, Taichung 40724, Taiwan; E-Mails: m0111617@fcu.edu.tw (C.-W.C.); p0156676@fcu.edu.tw (H.-Y.T.)

**Keywords:** diamino-substituted perylene tetracarboxylic dianhydrides, intramolecular charge transfer, near-infrared fluorescent dyes, solvatochromism, Lippert–Mataga equation, density functional theory calculations

## Abstract

1,6- and 1,7-regioisomers of diamino-substituted perylene tetracarboxylic dianhydrides (PTCDs) with different *n*-alkyl chain lengths (n = 6, 12 or 18) were synthesized and characterized by NMR spectroscopy and high-resolution mass spectrometry. These dyes are highly soluble in most organic solvents and even in nonpolar solvents, such as hexane. To the best of our knowledge, this is the first time the 1,6-diamino-substituted PTCDs (**2a**–**2c**) have been obtained in pure form. The regioisomers **1a**–**1c** (1,7-) and **2a**–**2c** (1,6-) exhibit significant differences in their optical characteristics. In addition to the longest wavelength absorption band at around 674 nm, **2a**–**2c** exhibit another shoulder band at *ca.* 600 nm, and consequently, cover a large part of the visible region relative to those of **1a**–**1c**. Upon excitation, **2a**–**2c** also show larger dipole moment changes than those of **1a**–**1c**; the dipole moments of all compounds have been estimated using Lippert–Mataga equation. Moreover, all the dyes show a unique charge transfer emission in the near-infrared region, of which the peak wavelengths exhibit strong solvatochromism. They all exhibit one irreversible one-electron oxidation and two quasi-reversible one-electron reductions in dichloromethane at modest potentials. Complementary density functional theory calculations performed on these chromophores are reported in order to rationalize their electronic structure and optical properties.

## 1. Introduction

Perylene diimides (PDIs) and perylene tetracarboxylic dianhydrides (PTCDs) have received significant attention in both academic and industrial research due to the favorable combination of excellent thermal and photostability, reversible redox properties, ease of synthetic modification, high molar absorptivities and photoluminescence quantum yields [1,2,3,4,5,6,7,8,9,10,11,12,13,14,15,16]. Due to these desirable attributes, PDIs and PTCDs have been used in a variety of applications in the field of organic electronics and optical devices, such as organic light-emitting diodes (OLEDs) [17,18], organic field-effect transistors (OFETs) [19,20], photochromic materials [21,22], molecular wires [23,24], LCD color filters [25,26], light-harvesting arrays [27,28] and organic solar cells (OSCs) [29,30,31,32]. PDIs and PTCDs have also been utilized in many other applications such as artificial photosynthetic systems through controlled supramolecular architectures via intermolecular π-π stacking [33,34]. As a result, more and more PDI and PTCD derivatives with interesting properties have been reported in the literature [35,36,37,38,39,40,41,42,43,44,45,46,47,48,49,50].

**Scheme 1:**
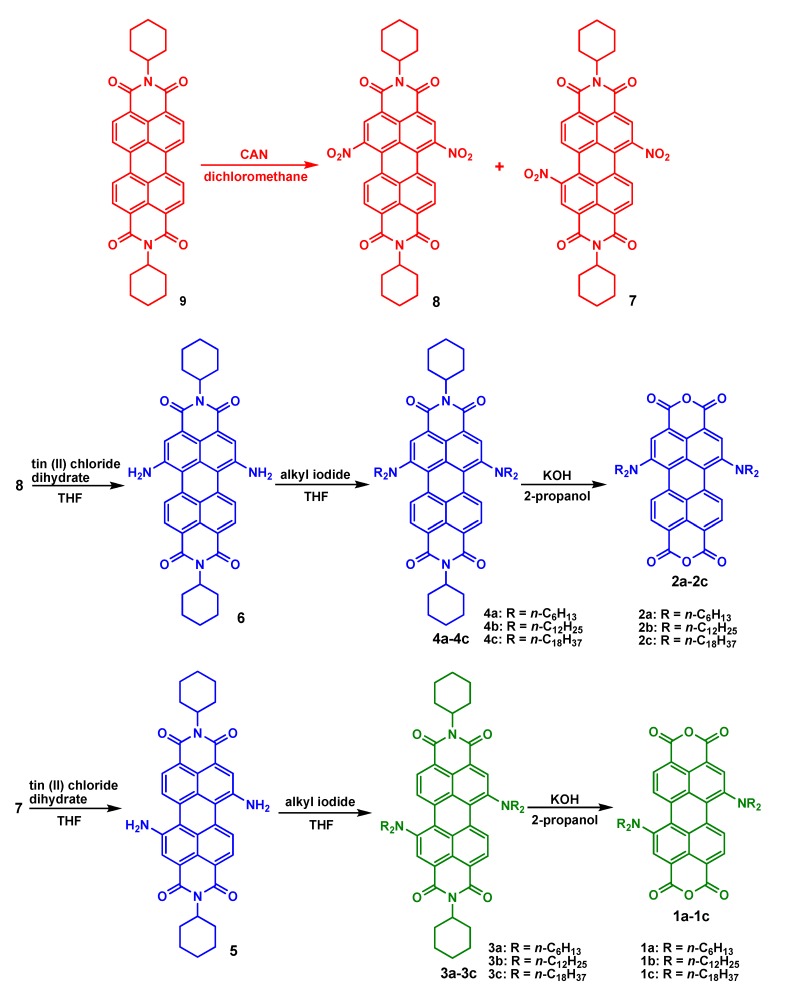
The synthetic routes for **1**–**4**.

PDIs and PTCDs suffer from serious problems, including aggregation and poor solubility. To overcome these drawbacks, several synthetic methods to prepare PDI and PTCD derivatives with improved solubility have been reported [43,44,47]. The synthesis of highly soluble PDIs and PTCDs is particularly important for process ability and for the preparation of their thin films to be used in organic electronics, such as OLEDs, OFETs and OSCs. Soluble PDI derivatives can be prepared by introducing long and bulky groups at the perylene core and/or at the imide nitrogen atoms, while soluble PTCD derivatives can only be obtained by introducing substituents at the perylene core. Thus, a number of 1,7-diamino-substituted PTCDs based on this method have been synthesized and studied so far [51,52,53,54]. However, to the best of our knowledge, the molecular structures, as well as the optical and electrochemical properties of 1,6-diamino-substituted PTCDs have not been reported yet. In an effort to expand the scope of highly soluble PTCD-based molecules available for designing systems for colorful dyes and photovoltaic cells, we herein report the detailed synthesis and characterization of 1,7- and 1,6-diamino-substituted PTCDs (**1a**–**1c** and **2a**–**2c**), shown in Scheme 1. The optical, electrochemical and complementary density functional theory (DFT) calculations of the newly synthesized PTCD dyes are also investigated.

## 2. Experimental Section

### 2.1. General

The starting materials, including perylene-3,4,9,10-tetracarboxyldianhydride, acetic acid, cyclohexylamine, cerium (IV) ammonium nitrate (CAN), tin (II) chloride dihydrate (SnCl_2_.2H_2_O), *N*-methyl-2-pyrrolidinone (NMP), tetrahydrofuran (THF), sodium hydride (NaH), 1-iodohexane (C_6_H_13_I, n = 6), 1-iodododecane (C_12_H_25_I, n = 12), and 1-iodooctadecane (C_18_H_37_I, n = 18) were purchased from Merck (Whitehouse Station, NJ, USA), ACROS (Pittsburgh, PA, USA) and Sigma-Aldrich (St. Louis, MO, USA). Solvents were distilled freshly according to standard procedure. Column chromatography was performed using silica gel Merck Kieselgel *si* 60 (40–63 mesh). ^1^H and ^13^C NMR spectra were recorded in CDCl_3_ on a Bruker 400 MHz NMR spectrometer (Bruker Corporation, Palo Alto, CA, USA). Mass spectra were recorded on a VG70-250S mass spectrometer (Hitachi, Ltd., Tokyo, Japan). The absorption and emission spectra were measured using a Jasco V-570 UV-Vis spectrophotometer and Hitachi F-7000 fluorescence spectrophotometer (Hitachi, Ltd., Tokyo, Japan), respectively. Cyclic voltammetry (CV) was performed with a CH instruments (CH INSTRUMENTS INC., Austin, TX, USA) at a potential rate of 200 mV·s^−1^ in a 0.1 M solution of tetrabutylammonium hexafluorophosphate (TBAPF_6_) in dichloromethane. Platinum and Ag/AgNO_3_ electrodes were used as counter and reference electrodes, respectively.

### 2.2. Synthesis

#### 2.2.1. Synthesis of 1,7- and 1,6-Dinitroperylene Diimides (**7** and **8**)

Compound **9** (1.0 g, 1.8 mmol), CAN (4.8 g, 8.8 mmol), nitric acid (8.0 g, 131.1 mmol) and dichloromethane (250 mL) were stirred at 25 °C under N_2_ for 48 h. The mixture was neutralized with 10% KOH and extracted with CH_2_Cl_2_. After solvent was removed, the crude product was purified by silica gel column chromatography with eluent CH_2_Cl_2_ to afford a mixture of 1,7- and 1,6-dinitroperylene diimides, and ^1^H-NMR (400 MHz) analysis revealed a 3:1 ratio. Separation of the 1,7 and 1,6 isomers was performed on a preparative HPLC system equipped with a refractive index detector and fitted with a macro-HPLC column (Si, 8 μm, 250 × 22 mm). The eluent was 8:1 hexane/ethyl acetate flowing at 12 mL·min^−1^. Two fractions were collected from the column; the first was pure 1,6 isomer (*R*_f_ = 0.42, 242 mg, yield = 21%), and the second was pure 1,7 isomer (*R*_f_ = 0.38, 682 mg, yield = 59%). Characterization data: **7**: ^1^H NMR (400 MHz, CDCl_3_) δ 8.78 (2H, s), 8.68 (2H, d, *J =* 8.4 Hz), 8.28 (2H, d, *J =* 8.4 Hz), 5.01 (2H, m), 2.51 (4H, m), 1.92 (4H, m), 1.74 (6H, m), 1.46 (4H, m), 1.36 (2H, m); MS (FAB): *m/z* (relative intensity) 645 [M+H^+^, 100]; HRMS calcd. for C_36_H_29_O_8_N_4_ 645.1985, found 645.1977. Selected data for **8**: ^1^H NMR (400 MHz, CDCl_3_) δ 8.78 (2H, s), 8.63 (2H, d, *J =* 8.0 Hz), 8.30 (2H, d, *J =* 8.0 Hz), 5.01 (2H, m), 2.52 (4H, m), 1.90 (4H, m), 1.74 (6H, m), 1.46 (4H, m), 1.36 (2H, m); MS (FAB): *m/z* (relative intensity) 645 [M+H^+^, 100]; HRMS calculated for C_36_H_29_O_8_N_4_ 645.1985, found 645.1983.

#### 2.2.2. Synthesis of 1,7- and 1,6-Diaminoperylene Diimides (**5** and **6**)

Tin (II) chloride dihydrate (1.0 g, 4.8 mmol), 1,7- or 1,6-dinitroperylene diimides (0.5 g, 0.8 mmol) were suspended in THF (50 mL), and stirred at 25 °C under N_2_ for 20 min. The solvent was refluxed 80 °C with stirring for 6 h. THF is removed at the rotary evaporator, and the residue was dissolved in ethyl acetate and washed with 10% sodium hydroxide solution and brine. The organic layer was dried over anhydrous MgSO_4_ and the filtrate was concentrated under reduced pressure. The crude product was purified by silica gel column chromatography with eluent ethyl acetate/*n*-hexane (4/5) to afford **5** (**6**) in 82% (80%) yield. Characterization data: **5**: ^1^H NMR (400 MHz, CDCl_3_) δ 8.90 (2H, d, *J* = 8.0 Hz), 8.25 (2H, d, *J* = 8.0 Hz), 8.14 (2H, s), 5.04, (2H, m), 4.94 (4H, s), 2.61 (4H, m), 1.93 (4H, m), 1.74 (6H, m), 1.36–1.54 (6H, m); MS (FAB): *m/z* (relative intensity) 585 (M+H^+^, 100); HRMS calcd. for C_36_H_33_O_4_N_4_ 585.2502, found 585.2504. Selected data for **6**: ^1^H NMR (400 MHz, CDCl_3_) δ 8.77 (2H, d, *J* = 8.0 Hz), 8.51 (2H, d, *J* = 8.0 Hz), 7.85 (2H, s), 5.05 (2H, m), 4.98 (4H, s), 2.59 (4H, m), 1.92 (4H, m), 1.76 (6H, m), 1.27–1.56 (6H, m); MS (FAB): *m/z* (relative intensity) 585 [M+H^+^, 100]; HRMS calcd. for C_36_H_33_O_4_N_4_ 585.2502, found 585.2508.

#### 2.2.3. General Procedure for Alkylation (**3a**–**3c** and **4a**–**4c**)

A mixture of solution of 1,7- or 1,6-diaminoperylene diimides (410 mg, 0.70 mmol), sodium hydride (97%, 200 mg, 8.00 mmol) and dry THF (60 mL) was stirred at 0 °C under N_2_ for 30 min. Alkyl iodide (4.20 mmol) was then added and the resulting mixture was stirred for 8 h. The resulting mixture was diluted with 15 mL of water and extracted with CH_2_Cl_2_. The crude product was purified by silica gel column chromatography with eluent ethyl acetate/n-hexane (1/2) to afford **3a** (**3b** or **3c**) or **4a** (**4b** or **4c**) in 75% yield. Characterization data: **3a**: ^1^H NMR (400 MHz, CDCl_3_) δ 9.21 (d, *J* = 8.0 Hz, 2H), 8.45 (s, 2H), 8.38 (d, *J* = 8.0 Hz, 2H), 5.03, (m, 2H), 3.45 (m, 4H), 3.15 (m, 4H), 2.57 (m, 4H), 1.87 (m, 4H), 1.15–1.75 (m, 44H), 0.82 (t, *J* = 6.4 Hz, 12H); ^13^C NMR (100 MHz, CDCl_3_) δ 164.5, 164.2, 148.5, 135.4, 130.3, 128.2, 125.3, 124.3, 122.9, 122.7, 122.6, 121.1, 53.8, 52.5, 31.4, 29.1, 27.5, 26.9, 26.6, 25.5, 22.5, 13.9; MS (FAB): m/z (relative intensity) 921 (M+H^+^, 100); HRMS calcd. for C_60_H_81_O_4_N_4_ 921.6256, found 921.6250. Selected data for **3b**: ^1^H NMR (400 MHz, CDCl_3_) δ 9.20 (d, *J* = 8.4 Hz, 2H), 8.45 (s, 2H), 8.37 (d, *J* = 8.4 Hz, 2H), 5.04, (m, 2H), 3.46 (m, 4H), 3.17 (m, 4H), 2.60 (m, 4H), 1.87 (m, 4H), 1.12–1.75 (m, 92H), 0.85 (t, *J* = 6.5 Hz, 12H); ^13^C NMR (100 MHz, CDCl_3_) δ 164.4, 164.1, 148.5, 135.3, 130.3, 128.1, 125.3, 124.2, 122.8, 122.7, 122.6, 121.1, 53.7, 52.4, 31.8, 29.5, 29.4, 29.2, 29.1, 29.0, 27.5, 27.2, 26.6, 25.5, 22.6, 14.0; MS (FAB): m/z (relative intensity) 1258 (M+H^+^, 100); HRMS calcd. for C_84_H_129_O_4_N_4_ 1258.0014, found 1258.0004. Selected data for **3c**: ^1^H NMR (400 MHz, CDCl_3_) δ 9.19 (d, *J* = 8.4 Hz, 2H), 8.45 (s, 2H), 8.38 (d, *J* = 8.4 Hz, 2H), 5.04, (m, 2H), 3.45 (m, 4H), 3.15 (m, 4H), 2.60 (m, 4H), 1.87 (m, 4H), 1.14–1.74 (m, 140H), 0.86 (t, *J* = 6.4 Hz, 12H); ^13^C NMR (100 MHz, CDCl_3_) δ 164.5, 164.1, 148.5, 135.4, 130.3, 128.2, 125.3, 124.3, 122.9, 122.8, 122.7, 121.1, 53.8, 52.5, 31.9, 29.7, 29.5, 29.3, 29.2, 29.1, 27.5, 27.2, 26.6, 25.5, 22.7, 14.1; MS (FAB): m/z (relative intensity) 1595 (M+H^+^, 100); HRMS calcd. for C_108_H_177_O_4_N_4_ 1595.3803, found 1595.3815. Selected data for **4a**: ^1^H NMR (400 MHz, CDCl_3_) δ 9.40 (d, *J* = 8.0 Hz, 2H), 8.55 (d, *J* = 8.0 Hz, 2H), 8.32 (s, 2H), 5.06, (m, 2H), 3.35 (m, 4H), 3.01 (m, 4H), 2.57 (m, 4H), 1.90 (m, 4H), 1.15–1.77 (m, 44H), 0.78 (t, *J* = 6.4 Hz, 12H); ^13^C NMR (100 MHz, CDCl_3_) δ 164.5, 164.3, 151.0, 135.8, 131.9, 130.9, 128.9, 128.0, 123.7, 123.2, 123.1, 123.0, 120.3, 54.0, 53.6, 52.8, 31.5, 29.2, 29.1, 27.3, 26.9, 26.6, 25.5, 22.5, 13.9; MS (FAB): m/z (relative intensity) 921 (M+H^+^, 100); HRMS calcd. for C_60_H_81_O_4_N_4_ 921.6256, found 921.6247. Selected data for **4b**: ^1^H NMR (400 MHz, CDCl_3_) δ 9.38 (d, *J* = 8.3 Hz, 2H), 8.55 (d, *J* = 8.3 Hz, 2H), 8.32 (s, 2H), 5.04, (m, 2H), 3.34 (m, 4H), 3.01 (m, 4H), 2.58 (m, 4H), 1.87 (m, 4H), 1.10–1.77 (m, 92H), 0.85 (t, *J* = 6.5 Hz, 12H); ^13^C NMR (100 MHz, CDCl_3_) δ 164.4, 164.2, 151.0, 135.8, 131.9, 130.9, 128.9, 128.0, 123.7, 123.1, 123.0, 120.3, 54.0, 53.6, 52.7, 31.9, 29.6, 29.5, 29.3, 29.1, 27.4, 27.3, 26.6, 25.5, 22.7, 14.1; MS (FAB): m/z (relative intensity) 1258 (M+H^+^, 100); HRMS calcd. for C_84_H_129_O_4_N_4_ 1258.0014, found 1258.0001. Selected data for **4c**: ^1^H NMR (400 MHz, CDCl_3_) δ 9.38 (d, *J* = 8.3 Hz, 2H), 8.55 (d, *J* = 8.4 Hz, 2H), 8.32 (s, 2H), 5.01, (m, 2H), 3.35 (m, 4H), 3.01 (m, 4H), 2.53 (m, 4H), 1.90 (m, 4H), 1.13–1.77 (m, 140H), 0.86 (t, *J* = 6.8 Hz, 12H); ^13^C NMR (100 MHz, CDCl_3_) δ 164.4, 164.2, 151.0, 135.8, 134.2, 131.9, 130.8, 129.4, 128.8, 128.0, 123.7, 123.1, 123.0, 120.3, 54.0, 53.6, 52.7, 31.9, 29.6, 29.5, 29.4, 29.3, 29.2, 27.3 27.2, 22.6, 14.1; MS (FAB): m/z (relative intensity) 1595 (M+H^+^, 100); HRMS calcd. for C_108_H_177_O_4_N_4_ 1595.3803, found 1595.3813.

#### 2.2.4. General Procedure for Saponification (**1a**–**1c** and **2a**–**2c**)

1,7- or 1,6-dialkylaminoperylene diimides (0.27 mmol) was taken in 2-propanol (30 mL) and subsequently KOH (1.9 g, 33.8 mmol) was added. The reaction mixture was stirred under N_2_ at reflux for 4 h. After being cooled to room temperature, the reaction mixture was poured into acetic acid (50 mL) and stirred overnight. The resulting green precipitate was collected by filtration, washed with water and methanol, and dried. The crude product was purified by silica gel column chromatography with eluent CH_2_Cl_2_ to afford **1a** (**1b** or **1c**) or **2a** (**2b** or **2c**) in 75% yield. Characterization data: **1a**: ^1^H NMR (400 MHz, CDCl_3_) δ 9.01 (d, *J* = 8.0 Hz, 2H), 8.44 (s, 2H), 8.37 (d, *J* = 8.0 Hz, 2H), 3.46 (m, 4H), 3.14 (m, 4H), 1.55 (m, 8H), 1.19 (m, 24H), 0.76 (t, *J* = 6.9 Hz, 12H); ^13^C NMR (100 MHz, CDCl_3_) δ 160.8 160.1, 149.0, 136.5, 130.9, 130.0, 127.1, 126.3, 123.0, 122.7, 118.5, 116.7, 52.6, 31.4, 27.6, 26.8, 22.4, 13.9; MS (FAB): m/z (relative intensity) 759 (M+H^+^, 100); HRMS calcd. for C_48_H_59_O_6_N_2_ 759.4373, found 759.4381. Selected data for **1b**: ^1^H NMR (400 MHz, CDCl_3_) δ 8.95 (d, *J* = 8.0 Hz, 2H), 8.42 (s, 2H), 8.34 (d, *J* = 8.0 Hz, 2H), 3.45 (m, 4H), 3.12 (m, 4H), 1.60 (m, 10H), 1.12–1.25 (m, 70H), 0.87 (t, *J* = 5.4 Hz, 12H); ^13^C NMR (100 MHz, CDCl_3_) δ 160.7 160.0, 148.9, 136.3, 130.8, 129.9, 127.0, 126.2, 122.8, 122.5, 118.4, 116.6, 52.5, 31.8, 29.6, 29.5, 29.4, 29.3, 29.2, 27.6, 27.1, 22.6, 14.0; MS (FAB): m/z (relative intensity) 1095 (M+H^+^, 100); HRMS calcd. for C_72_H_107_O_6_N_2_ 1095.8129, found 1095.8115. Selected data for **1c**: ^1^H NMR (400 MHz, CDCl_3_) δ 9.01 (d, *J* = 8.3 Hz, 2H), 8.45 (s, 2H), 8.38 (d, *J* = 8.3 Hz, 2H), 3.46 (m, 4H), 3.14 (m, 4H), 1.57 (m, 10H), 1.15–1.22 (m, 118H), 0.85 (t, *J* = 6.6 Hz, 12H); ^13^C NMR (100 MHz, CDCl_3_) δ 160.8, 160.1, 148.9, 136.5, 130.9, 130.0, 127.1, 126.3, 123.0, 122.7, 118.5, 116.8, 52.6, 31.9, 29.7, 29.5, 29.4, 29.3, 29.2, 27.6, 27.2, 22.7, 14.1; MS (FAB): m/z (relative intensity) 1433 (M+H^+^, 100); HRMS calcd. for C_96_H_155_O_6_N_2_ 1433.1919, found 1433.1903. Selected data for **2a**: ^1^H NMR (400 MHz, CDCl_3_) δ 9.20 (d, *J* = 8.4 Hz, 2H), 8.58 (d, *J* = 8.4 Hz, 2H), 8.31 (s, 2H), 3.40 (m, 4H), 3.01 (m, 4H), 1.64 (m, 4H), 1.48 (m, 4H), 1.13–1.17 (m, 24H), 0.82 (t, *J* = 6.8 Hz, 12H); ^13^C NMR (100 MHz, CDCl_3_) δ 160.6 160.5, 151.8, 137.1, 133.0, 132.5, 131.6, 128.5, 125.1, 123.0, 122.8, 121.6, 119.2, 115.6, 52.8, 31.4, 27.5, 26.8, 22.5, 13.9; MS (FAB): m/z (relative intensity) 759 (M+H^+^, 100); HRMS calcd. for C_48_H_59_O_6_N_2_ 759.4373, found 759.4379. Selected data for **2b**: ^1^H NMR (400 MHz, CDCl_3_) δ 9.20 (d, *J* = 8.4 Hz, 2H), 8.58 (d, *J* = 8.4 Hz, 2H), 8.31 (s, 2H), 3.39 (m, 4H), 3.02 (m, 4H), 1.65 (m, 4H), 1.59 (m, 4H), 1.13–1.17 (m, 72H), 0.82 (t, *J* = 5.4 Hz, 12H); ^13^C NMR (100 MHz, CDCl_3_) δ 160.5, 160.4, 151.9, 137.1, 133.0, 132.5, 131.6, 128.5, 125.1, 123.0, 122.8, 121.6, 119.2, 115.7, 52.8, 31.9, 29.6, 29.5, 29.4, 29.3, 27.5, 27.2, 22.6, 14.1; MS (FAB): m/z (relative intensity) 1095 (M+H^+^, 100); HRMS calcd. for C_72_H_107_O_6_N_2_ 1095.8129, found 1095.8113. Selected data for **2c**: ^1^H NMR (400 MHz, CDCl_3_) δ 9.20 (d, *J* = 8.4 Hz, 2H), 8.58 (d, *J* = 8.3 Hz, 2H), 8.31 (s, 2H), 3.39 (m, 4H), 3.00 (m, 4H), 1.47–1.60 (m, 8H), 1.14–1.21 (m, 120H), 0.84 (t, *J* = 6.4 Hz, 12H); ^13^C NMR (100 MHz, CDCl_3_) δ 160.5, 160.4, 151.8, 137.1, 132.9, 132.4, 131.5, 128.5, 125.0, 122.9, 122.7, 121.6, 119.1, 115.6, 52.7, 31.9, 29.6, 29.5, 29.4, 29.3, 29.2, 29.1, 27.5, 27.1, 22.6, 14.1; MS (FAB): m/z (relative intensity) 1433 (M+H^+^, 100); HRMS calcd. for C_96_H_155_O_6_N_2_ 1433.1919, found 1433.1905.

## 3. Results and Discussion

### 3.1. Synthesis

Scheme 1 shows the chemical structures and synthetic routes of 1,7- and 1,6-dialkylamino-substituted PTCDs (**1a**–**1c** and **2a**–**2c**). The synthesis of **1a**–**1c** and **2a**–**2c** started from a dinitration of perylene diimide **9** [48], giving dinitroperylene diimides in 80% yield. Among the products, a 3:1 mixture of regioisomers (nitrated at the 1,7- or 1,6-positions) was observed by ^1^H-NMR spectroscopy [48]. The regioisomeric 1,7- and 1,6-dinitroperylene diimides (**7** and **8**) can be separated by high performance liquid chromatography (HPLC). Subsequently, the reduction of **7** (**8**) by tin (II) chloride dihydrate (SnCl_2_.2H_2_O) in refluxing THF obtained **5** (**6**). Next, three highly soluble alkylamino-substituted PDI derivatives **3a**–**3c (4a**–**4c)** with different *N*-alkyl chain lengths (*n*-C_6_H_13_, *n*-C_12_H_25_ or *n*-C_18_H_37_) were prepared by the alkylation of **5** (**6**) with the corresponding alkyl halides. Finally, alkylamino-substituted PDIs **3a**–**3c** (**4a**–**4c**) were converted to the respective PTCDs via saponification to afford **1a**–**1c** (**2a**–**2c**). Detailed synthetic procedures and product characterization are provided in the Experimental Section and Supplementary Materials (Figures S1–S24).

**Figure 1:**
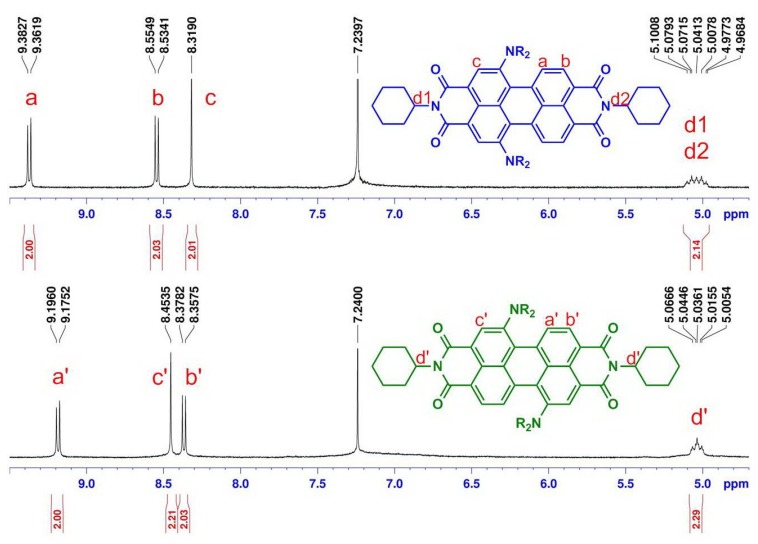
^1^H NMR (400 MHz, CDCl_3_) partial spectra of regioisomerically pure perylene bisimides **3a** (bottom) and **4a** (top).

It is to be noted that the characteristic signals of the regioisomers **3a** (1,7-) and **4a** (1,6-) in the ^1^H NMR spectra (Figure 1), one singlet and two doublets of perylene core protons, exhibit significant differences in the chemical shift values (0.18 and 0.19 ppm for the doublets at 8.30–9.40). The aromatic signals for **3a** (1,7-) appear in different order (doublet, singlet, doublet) compared to that of **4a** (1,6-) (doublet, doublet, singlet). This different pattern of appearance of singlet and doublets makes them easily recognizable by 400 MHz ^1^H-NMR. More importantly, a convenient unequivocal assignment of the NMR spectrum to the individual regioisomers **3a** (1,7-) and **4a** (1,6-) was performed on the basis of the signal of methine protons next to the imide nitrogen at 5.04 ppm. Because of the same chemical environment, both two methine protons of major regioisomer **3a** (1,7-) appear as one common multiplet at 5.04 ppm, but the signal splits into double multiplets for minor regioisomer **4a** (1,6-). In this way, an unambiguous characterization has been made successfully on the basis of 400 MHz ^1^H NMR.

### 3.2. Optical Properties

**Figure 2:**
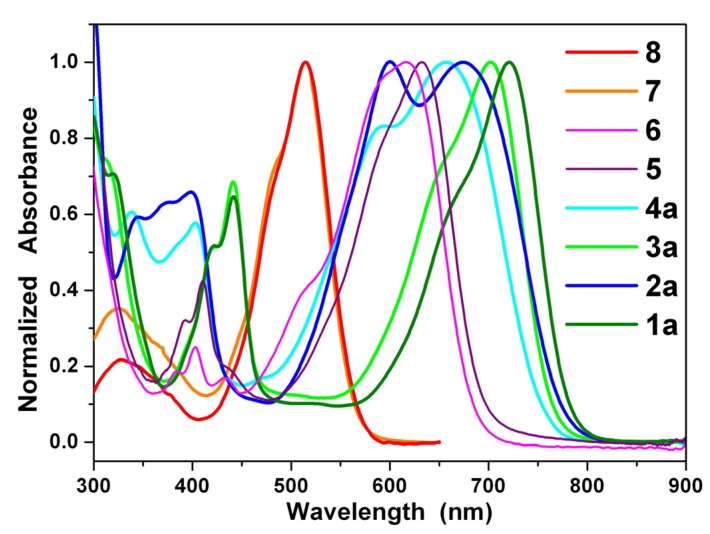
Normalized absorption spectra of **1a**–**4a** and **5**–**8** in dichloromethane solution.

Figure 2 shows the steady state absorption spectra of the green dyes **1a** and **3a**, the blue dyes **2a**, **4a**, **5**, and **6**, and the red dyes **7** and **8** in dichloromethane. The spectra of **1b**, **1c**, **2b**, and **2c** can be found in the Supplementary Materials (Figures S25–S28). The absorption spectra of 1,7- and 1,6-dinitroperylene diimides (**7** and **8**) are nearly identical with the spectrum of the non-substituted perylene diimide (**9**), but they are non-fluorescent [48]. The reduction of **7** (**8**) to **5** (**6**) switches the substituents from strong electron-withdrawing nitro groups to electron-donating amino groups and causes a significant red shift [42]. The spectra of **1**–**6** are dominated by very broad absorption bands that cover a large part of the visible spectrum (300–800 nm). These broad bands are typical for perylene diimide (dianhydride) derivatives *N*-substituted at the bay-core positions, due to charge transfer absorption [43]. The longest wavelength absorption band of 1,7- and 1,6-diaminoperylene diimides (**5** and **6**) is red-shifted relative to that of 1,7- and 1,6-dinitroperylene diimides (**7** and **8**), but it is blue-shifted relative to that of 1,7- and 1,6-dialkylaminoperylene diimides (**3** and **4**). It appears that the inductive effect of the alkyl groups in **3** and **4** causes an additional red shift. The regioisomers **1a**–**1c** (1,7-) and **2a**–**2c** (1,6-) exhibit significant differences in their optical characteristics. In addition to the longest wavelength absorption band at around 674 nm, **2a**–**2c** exhibit another shoulder band at *ca.* 600 nm, and consequently, cover a large part of the visible region relative to those of **1a**–**1c**. Further careful examination of the absorption spectra of 1,6-disubstituted (**2**, **4** and **6**) and 1,7-disubstituted PDIs and PTCDs (**1**, **3** and **5**) also reveals that 1,6-disubstituted PDIs and PTCDs (**2**, **4** and **6**) cover a larger portion of the visible region (450–800 nm) compared to those of the corresponding 1,7-disubstituted PDIs and PTCDs (**1**, **3** and **5**). Interestingly, the longest wavelength absorption band of **1a** (1,7-) is 41 nm red-shifted relative to that of **2a** (1,6-); the decrease in the energy band gap is attributed to an increase in the HOMO energy level (*vide infra*). In addition, the longest wavelength absorption band of **1a**–**1c** and **2a**–**2c** exhibits a red shift when the solvent polarity increases (Table 1 and Table 2 and Figures S29 and S30), which is consistent with previous studies [43].

**Table 1:** Summary of optical absorption and emission properties of **1a**–**1c** in various solvents.

1a/1b/1c	λ_abs_ (nm) ^a^	log *ε* ^b^	λ_em_ (nm) ^a^	Stokes shift (nm)	Φ ^c^ × 10^2^
cyclohexane	678/679/679	4.67/4.66/4.64	717/716/716	39/37/37	4.31/3.71/3.32
diethyl ether	683/686/686	4.67/4.66/4.64	732/733/732	49/47/46	0.52/0.51/0.62
ethyl acetate	698/698/699	4.67/4.66/4.64	753/753/755	55/55/56	0.14/0.13/0.12
dichloromethane	713/714/714	4.68/4.67/4.65	775/771/773	59/57/58	0.13/0.24/0.26
acetonitrile	714/714/713	4.66/4.66/4.64	794/792/791	80/78/78	0.11/0.15/0.16

^a^ Measured at 2 × 10^−5^ M; ^b^ *ε* stands for extinction coefficient (in M^−1^·cm^−1^); ^c^ Determined with *N*,*N*’-dioctyl-3,4,9,10-perylenedicarboximide as reference [42].

**Table 2:** Summary of optical absorption and emission properties of **2a**–**2c** in various solvents.

2a/2b/2c	λ_abs_ (nm) ^a^	*ε* ^b^ (M^−1^·cm^−1^)	λ_em_ (nm) ^a^	Stokes shift (nm)	Φ ^c^ × 10^3^
cyclohexane	632/632/633	4.69/4.69/4.68	721/721/719	89/89/86	2.28/2.87/2.86
diethyl ether	634/639/641	4.69/4.69/4.68	735/736/732	101/97/91	0.48/0.38/0.65
ethyl acetate	652/649/653	4.69/4.68/4.68	760/755/757	108/106/101	0.27/0.32/0.40
dichloromethane	672/671/674	4.70/4.69/4.67	791/787/792	119/116/118	0.20/0.18/0.23
acetonitrile	667/671/670	4.69/4.68/4.67	800/799/799	133/128/129	0.15/0.17/0.19

^a^ Measured at 2 × 10^−5^ M; ^b^ *ε* stands for extinction coefficient (in M^−1^·cm^−1^); ^c^ Determined with *N*,*N*’-dioctyl-3,4,9,10-perylenedicarboximide as reference [42].

**Figure 3:**
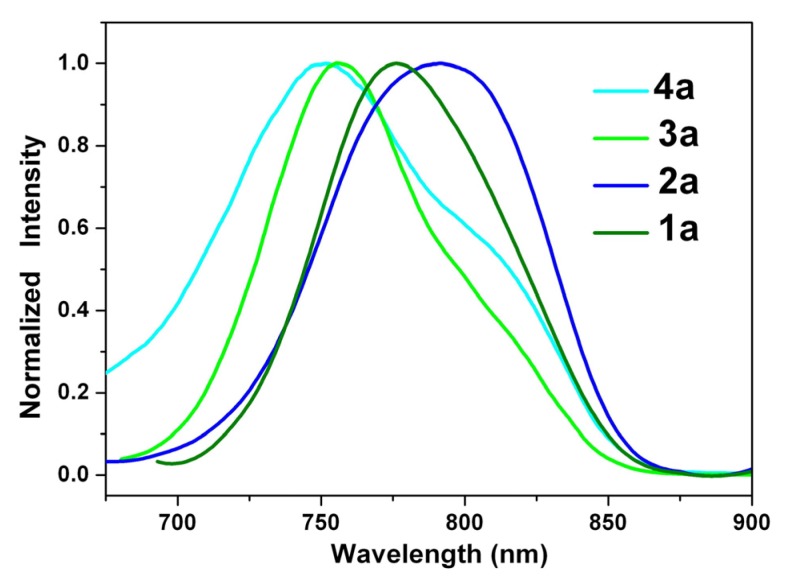
Normalized emission spectra of **1a**–**4a** in dichloromethane solution.

**Figure 4:**
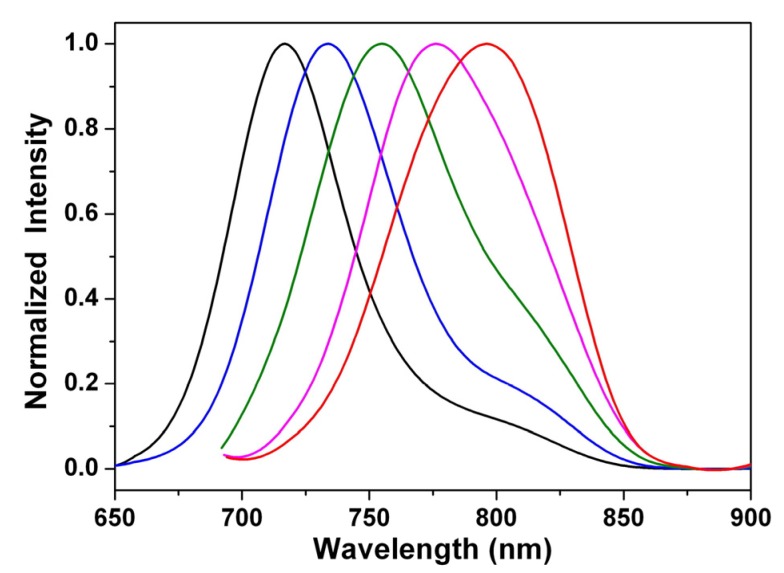
Normalized emission spectra of **1a** in cyclohexane (black line), diethyl ether (blue line), ethyl acetate (olive line), dichloromethane (pink line), and acetonitrile (red line).

**Figure 5:**
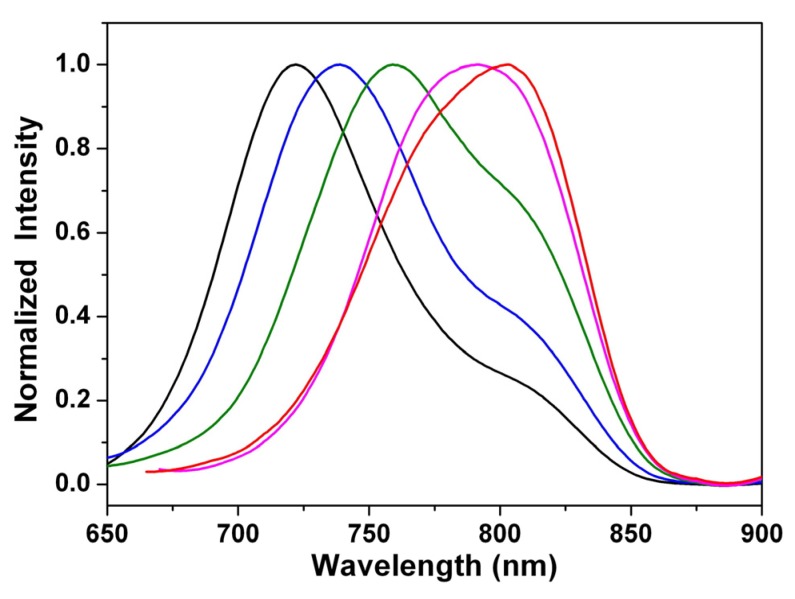
Normalized emission spectra of **2a** in cyclohexane (black line), diethyl ether (blue line), ethyl acetate (olive line), dichloromethane (pink line), and acetonitrile (red line).

**Figure 6:**
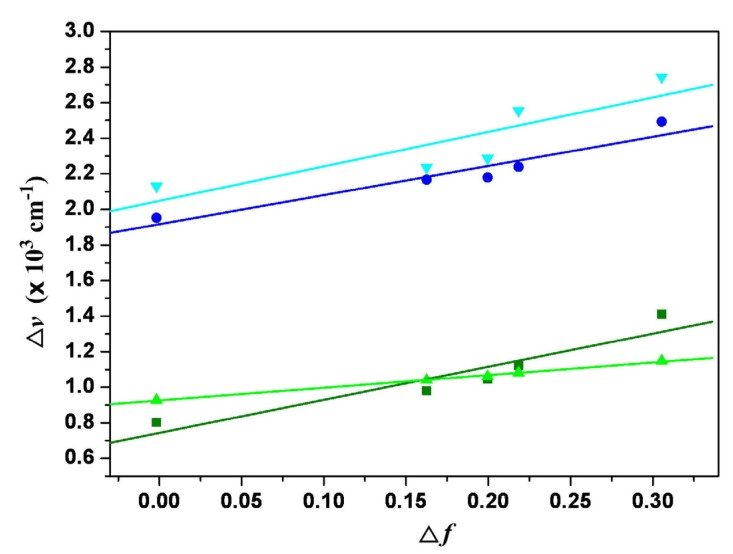
Lippert–Mataga plots for **1a** (olive line), **2a** (blue line), **3a** (green line), and **4a** (cyan line). The solvents from left to right are (1) cyclohexane, (2) diethyl ether, (3) ethyl acetate, (4) dichloromethane, (5) acetonitrile.

The fluorescence spectra of 1,7- and 1,6-dialkylamino-substituted PDIs (**3a** and **4a**) and PTCDs (**1a** and **2a**) in dichloromethane are shown in Figure 3; they all emit in the near-infrared region. Unlike the small shift in absorption spectra, the fluorescence spectra of **1**–**4** are largely red-shifted if there is any increase of the solvent polarity (Table 1 and Table 2, Table S1 and S2), which indicates strong intramolecular charge transfer (ICT) characteristics for the excited states of **1**–**4** (Figure 4, Figure 5 and Figure S31–S34). For instance, the shift of the emission peak of **1a** is 77 nm from cyclohexane (717 nm) to acetonitrile (794 nm), and that of **2a** is 79 nm. We used the well-established fluorescence solvatochromic shift method [55] to measure the stabilization of the excited states of **1a**–**4a**. The change of magnitudes for dipole moments between ground and excited states, *i*.*e.*, $\text{Δ}\mu =|\vec{\mu_{e}}-\vec{\mu_{g}}|$, can be calculated by the Lippert–Mataga equation and expressed as:

(1) $${\bar{\upsilon }}_{a}-{\bar{\upsilon }}_{f}=\frac{2}{hc}{\left(\mu_{e}-\mu_{g}\right)}^{2}{a_{0}}^{-3}\text{Δ}f+const.$$

where *h* is the Planck constant, *c* is the speed of light, and $a_{0}$ denotes the cavity radius in which the solute resides, ${\bar{\upsilon }}_{a}-{\bar{\upsilon }}_{f}$ is the Stokes shift of the absorption and emission peak maximum, and Δf is the orientation polarizability defined as:

(2) $$\text{Δ}f=f\left(\varepsilon \right)-f\left(n^{2}\right)=\frac{\varepsilon -1}{2\varepsilon +1}-\frac{n^{2}-1}{2n^{2}+1}$$

where *ε* and *n* are the static dielectric constant and the refractive index of the solvent, respectively. The plot of the Stokes shift ${\bar{\upsilon }}_{a}-{\bar{\upsilon }}_{f}$ as a function of Δf is sufficiently linear for **1a**–**4a** (Figure 6). Accordingly, $\text{Δ}\mu =|\vec{\mu_{e}}-\vec{\mu_{g}}|$ values can be estimated as 7.7 D, 11.7 D, 7.9 D, and 12.7 D for **1a**–**4a**. The results show that 1,6-disubstituted PDIs and PTCDs (**2** and **4**) have larger dipole moment changes than those of the corresponding 1,7-disubstituted PDIs and PTCDs (**1** and **3**).

### 3.3. Quantum Chemistry Computation

**Figure 7:**
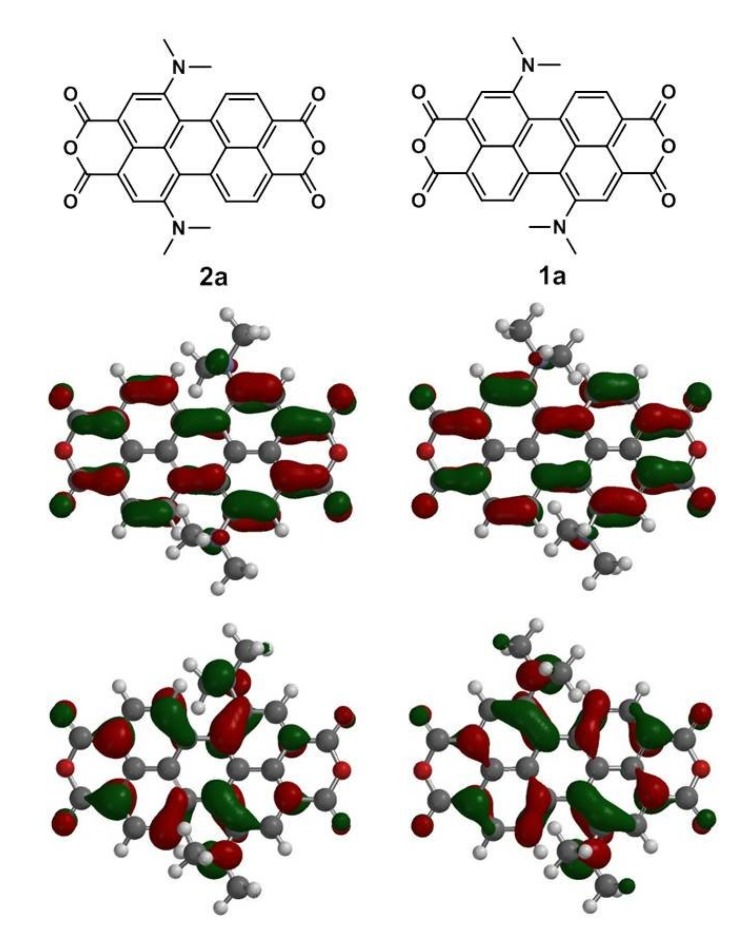
Calculated frontier orbitals for **1a** and **2a**. The upper structures show the LUMOs and the lower ones show the HOMOs. Methyl groups replace the hexyl groups for clarity.

To gain more insight into the molecular structures and electronic properties of 1,7- and 1,6-diamino-substituted PTCDs (**1a**–**1c** and **2a**–**2c**), quantum chemical calculations were performed using density functional theory (DFT) at the B3LYP/6-31G^**^ level. Figure 7 depicts the highest occupied molecular orbitals (HOMOs) and the lowest unoccupied molecular orbitals (LUMOs) of **1a** and **2a**. The HOMO of both **1a** and **2a** is delocalized mainly on the amino group and the perylene core, while the LUMO is extended from the central perylene core to the dianhydride groups. Table 3 summarizes the calculated and experimental parameters for perylene bisimide derivatives **1a**–**1c** and **2a**–**2c**. One can clearly see that the HOMO energy levels of **1a**–**1c** are slightly higher than those of **2a**–**2c**. The results demonstrate that the removal of one electron from **2a**–**2c** (1,6-) is more difficult in comparison to **1a**–**1c** (1,7-), which is consistent with the experimental results (vied infra). Moreover, the calculated HOMO–LUMO band gap energies of **1a**–**1c** and **2a**–**2c** are in good agreement with the experimental data.

**Figure 8:**
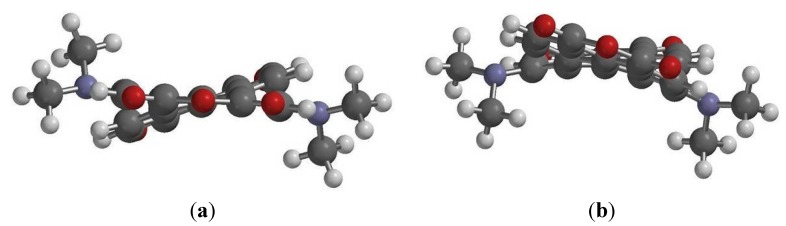
DFT (B3LYP/6-31G**) geometry-optimized structures of **1a** (**b**); and **2a** (**a**) shown with view along the long axis. Methyl groups replace the hexyl groups for clarity.

DFT calculations also indicate that the ground-state geometries of the perylene core have different core twist angles (Figure 8 and Table 3), *i*.*e*., approximate dihedral angles between the two naphthalene subunits attached to the central benzene ring; these are ~17.21° and ~17.30° for **1a**, ~17.23° and ~17.33° for **1b**, ~17.26° and ~17.35° for **1c**, ~19.36° and ~19.56° for **2a**, ~19.38° and ~19.59° for **2b** and ~19.41° and ~19.61° for **2c**. As a whole, the core twist angles of the 1,6-diamino-substituted PTCDs (**2a**–**2c**) are slightly larger than those of the 1,7-diamino-substituted PTCDs (**1a**–**1c**).

**Table 3:** Calculated and experimental parameters for 1,7- and 1,6-diamino-substituted PTCDs.

Compound	HOMO ^a^	LUMO ^a^	*E*_g_^a^	*E*_g_^b^	Twisting angle (°) ^a^
**1a**	−5.63	−3.52	2.11	1.83	17.21, 17.30
**1b**	−5.62	−3.52	2.10	1.83	17.23, 17.33
**1c**	−5.62	−3.52	2.10	1.83	17.26, 17.35
**2a**	−5.70	−3.51	2.19	1.96	19.36, 19.56
**2b**	−5.70	−3.51	2.19	1.96	19.38, 19.59
**2c**	−5.69	−3.51	2.18	1.96	19.41, 19.61

^a^ Calculated by DFT/B3LYP (in eV); ^b^ At absorption maxima (*E*_g_ = 1240/λ_max_, in eV).

### 3.4. Electrochemical Properties

**Figure 9:**
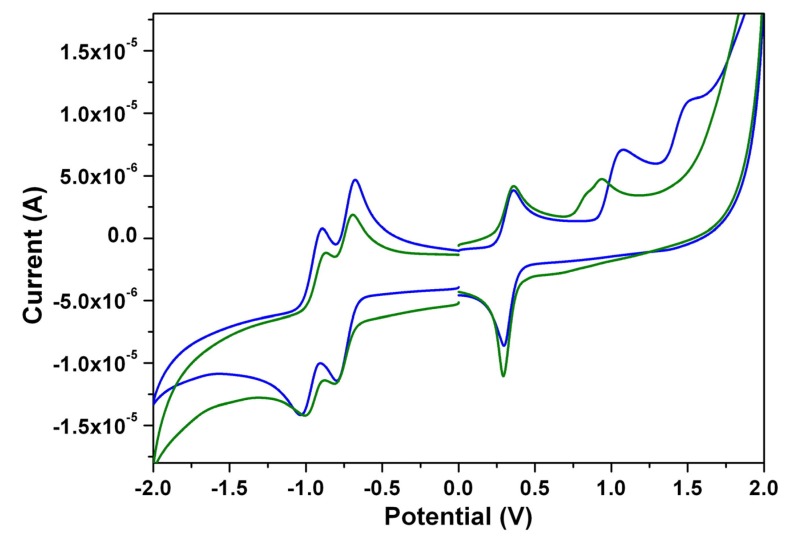
The cyclic voltammograms of **1a** (olive line) and **2a** (blue line) measured in dichloromethane solution with ferrocenium/ferrocene as an internal standard, at 200 mV·s^−1^.

The cyclic voltammograms of 1,7- and 1,6-diamino-substituted PTCDs (**1a** and **2a**) are illustrated in Figure 9. Both show one irreversible one-electron oxidation and two quasi-reversible one-electron reductions in dichloromethane. The one-electron oxidation of **1a** occurs at 0.95 V, whereas for **2a**, the first oxidation is slightly shifted to more positive values by 0.08 V. The results clearly indicate that the removal of one electron from **2a** (1,6-) is more difficult in comparison to **1a** (1,7-); these findings are in good agreement with previous reports [37]. Table 4 summarizes the redox potentials and the HOMO and LUMO energy levels estimated from cyclic voltammetry (CV) for **1a**–**1c** and **2a**–**2c**. The HOMO/LUMO energy levels of **1a**, **1b**, **1c**, **2a**, **2b**, and **2c** estimated to be −5.56/−3.73, −5.55/−3.72, −5.54/−3.71, −5.64/−3.68, −5.63/−3.67, and −5.63/−3.67 eV, respectively. The HOMO–LUMO energy gaps of **1a**–**1c** (**2a**–**2c**) are found to be almost the same, which indicates that different *N*-alkyl chain lengths do not significantly affect the band gap energies.

**Table 4:** Summary of half-wave redox potentials, HOMO and LUMO energy levels for 1,7- and 1,6-diamino-substituted PTCDs.

Compound	*E*^+^_1/2_^a^	*E*^-^_1/2_^a^	*E*^2-^_1/2_^a^	HOMO ^b^	LUMO ^b^
**1a**	0.95	−0.80	−1.02	−5.56	−3.73
**1b**	0.94	−0.81	−1.03	−5.55	−3.72
**1c**	0.93	−0.83	−1.03	−5.54	−3.71
**2a**	1.03	−0.81	−1.01	−5.64	−3.68
**2b**	1.02	−0.82	−1.02	−5.63	−3.67
**2c**	1.02	−0.83	−1.03	−5.63	−3.67

^a^ Measured in a solution of 0.1 M tetrabutylammonium hexafluorophosphate (TBAPF_6_) in dichloromethane *versus* SCE (in V); ^b^ Calculated from *E*_HOMO_ = −4.88 − (*E*_oxd_ − *E*_Fc/Fc+_), *E*_LUMO_ = *E*_HOMO_ + *E*_g_.

### 3.5. Stacking Behaviors of Dyes in Solution and Solid State

**Figure 10:**
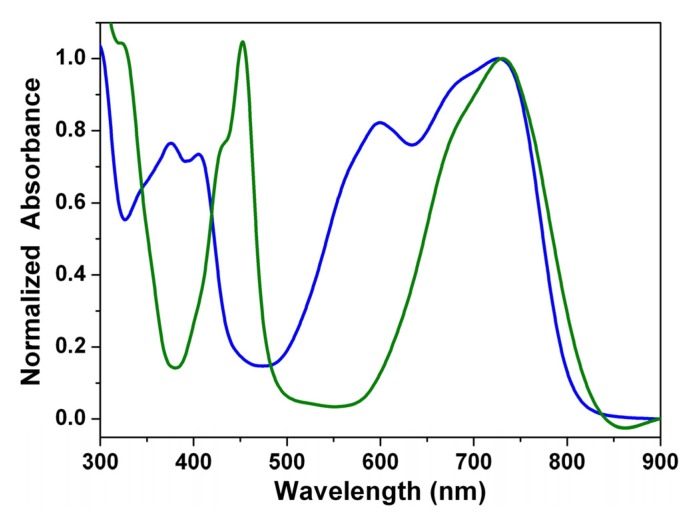
Normalized absorption spectra of **1a** and **2a** in neat film.

Figure 10 shows the absorption spectra recorded for thin drop-cast films of **1a** and **2a**. The shapes of the absorption spectra of **1a** and **2a** in solution (Figure 2) and in solid state are found to be virtually the same in view of wavelength range (300–800 nm) and peak positions, which demonstrates that it is difficult for **1a** and **2a** to form π-aggregates. Therefore, we can ascertain that the long alkyl chains not only largely increases the solubility of 1,7- and 1,6-diamino-substituted PTCDs (**1** and **2**), but also efficiently reduces intermolecular contact and aggregation.

## 4. Conclusions

We have successfully synthesized 1,7- and 1,6-diamino-substituted perylene tetracarboxylic dianhydrides (PTCDs) with different *n*-alkyl chain lengths (**1a**–**1c** and **2a**–**2c**). The dyes are soluble in most organic solvents and even in nonpolar solvents, such as hexane. To the best of our knowledge, this is the first time the 1,6-diamino-substituted PTCDs (**2a**–**2c**) have been obtained in pure form. Our studies have also shown that the 1,7- and 1,6-isomers can readily be characterized by 400 MHz ^1^H NMR. The regioisomers **1a**–**1c** (1,7-) and **2a**–**2c** (1,6-) exhibit significant differences in their optical characteristics. In addition to the longest wavelength absorption band at around 674 nm, **2a**–**2c** exhibit another shoulder band at ca. 600 nm, and consequently, cover a large part of the visible region relative to those of **1a**–**1c**. Upon excitation, **2a**–**2c** also show larger dipole moment changes than those of **1a**–**1c**; the dipole moments of all compounds have been estimated using Lippert–Mataga equation. All of the compounds **1a**–**1c** and **2a**–**2c** show a unique charge transfer emission in the near-infrared region, of which the peak wavelengths exhibit strong solvatochromism. Additionally, they undergo one irreversible one-electron oxidation and two quasi-reversible one-electron reductions in dichloromethane at modest potentials. Research on their applications to dye-sensitized solar cells (DSSCs) is currently in progress.

## Supplementary Materials

Supplementary materials can be accessed at: http://www.mdpi.com/1996-1944/8/8/4943/s1.

## References

[1] Langhals H., Kirner S. (2000). Novel fluorescent dyes by the extension of the core of perylenetetracarboxylic bisimides. Eur. J. Org. Chem..

[2] Liang Y., Wang H., Wang D., Liu H., Feng S. (2012). The synthesis, morphology and liquid-crystalline property of polysiloxane-modified perylene derivative. Dyes Pigment..

[3] Kaur B., Quazi N., Ivanov I., Bhattacharya S.N. (2012). Near-infrared reflective properties of perylene derivatives. Dyes Pigment..

[4] Daimon T., Nihei E. (2013). Fabrication of a poly(3-octylthiophene-2,5-diyl) electrochemiluminescence device assisted by perylene. Materials.

[5] Cui Y., Wu Y., Liu Y., Yang G., Liu L., Fu H., Li Z., Wang S., Wang Z., Chen Y. (2013). PEGylated nanoparticles of diperylene bisimides with high efficiency of ^1^O_2_ generation. Dyes Pigment..

[6] Wang R., Shi Z., Zhang C., Zhang A., Chen J., Guo W., Sun Z. (2013). Facile synthesis and controllable bromination of asymmetrical intermediates of perylene monoanhydride/monoimide diester. Dyes Pigment..

[7] Luo M.H., Chen K.Y. (2013). Asymmetric perylene bisimide dyes with strong solvatofluorism. Dyes Pigment..

[8] Kang H., Jiang W., Wang Z. (2013). Construction of well-defined butadiynylene-linked perylene bisimide arrays via cross-coupling. Dyes Pigment..

[9] Sharma G.D., Kurchania R., Ball R.J., Roy M.S., Mikroyannidis J.A. (2012). Effect of deoxycholic acid on the performance of liquid electrolyte dye-sensitized solar cells using a perylene monoimide derivative. Int. J. Photoenergy.

[10] Tsai H.Y., Chang C.W., Chen K.Y. (2014). 1,6- and 1,7-Regioisomers of asymmetric and symmetric perylene bisimides: synthesis, characterization and optical properties. Molecules.

[11] Tsai H.Y., Chen K.Y. (2014). Synthesis and optical properties of novel asymmetric perylene bisimides. J. Lumin..

[12] El-Daly S.A., Alamry K.A., Asiri A.M., Hussein M.A. (2012). Spectral characteristics and fluorescence quenching of *N*,*N*′-bis(4-pyridyl)-3,4:9,10-perylenebis(dicarboximide) (BPPD). J. Lumin..

[13] Naveenraj S., Raj M.R., Anandan S. (2012). Binding interaction between serum albumins and perylene-3,4,9,10-tetracarboxylate—A spectroscopic investigation. Dyes Pigment..

[14] Zhang L., Wang Y., Yu J., Zhang G., Cai X., Wu Y., Wang L. (2013). A colorimetric and fluorescent sensor based on PBIs for palladium detection. Tetrahedron Lett..

[15] Boobalan G., Imran P.M., Ramkumar S.G., Nagarajan S. (2014). Fabrication of luminescent perylene bisimide nanorods. J. Lumin..

[16] Tsai H.Y., Chang C.W., Lin C.W., Chen K.Y. (2015). 1,6- and 1,7-Regioisomers of dicyano-substituted perylene bisimides: synthesis, optical and electrochemical properties. J. Chin. Chem. Soc..

[17] Damaceanu M.D., Constantin C.P., Bruma N., Pinteala M. (2013). Tuning of the color of the emitted light from new polyperyleneimides containing oxadiazole and siloxane moieties. Dyes Pigment..

[18] Lucenti E., Botta C., Cariati E., Righetto S., Scarpellini M., Tordin E., Ugo R. (2013). New organic-inorganic hybrid materials based on perylene diimide-polyhedral oligomeric silsesquioxane dyes with reduced quenching of the emission in the solid state. Dyes Pigmtent..

[19] Jones B.A., Ahrens M.J., Yoon M.H., Facchetti A., Marks T.J., Wasielewski M.R. (2004). High-mobility air-stable *n*-type semiconductors with processing versatility: Dicyanoperylene-3,4:9,10-bis(dicarboximides). Angew. Chem. Int. Ed..

[20] Würthner F., Stolte M. (2011). Naphthalene and perylene diimides for organic transistors. Chem. Commun..

[21] Berberich M., Krause A.M., Orlandi M., Scandola F., Würthner F. (2008). Toward fluorescent memories with nondestructive readout: Photoswitching of fluorescence by intramolecular electron transfer in a diaryl ethene-perylene bisimide photochromic system. Angew. Chem. Int. Ed..

[22] Tan W., Li X., Zhang J., Tian H. (2011). A photochromic diarylethene dyad based on perylene diimide. Dyes Pigment..

[23] Weiss E.A., Ahrens M.J., Sinks L.E., Gusev A.V., Ratner M.A., Wasielewski M.R. (2004). Making a molecular wire: Charge and spin transport through para-phenylene oligomers. J. Am. Chem. Soc..

[24] Wilson T.M., Tauber M.J., Wasielewski M.R. (2009). Toward an *n*-type molecular wire: Electron hopping within linearly linked perylenediimide oligomers. J. Am. Chem. Soc..

[25] Choi J., Lee W., Sakong C., Yuk S.B., Park J.S., Kim J.P. (2012). Facile synthesis and characterization of novel coronene chromophores and their application to LCD color filters. Dyes Pigment..

[26] Sakong C., Kim Y.D., Choi J.H., Yoon C., Kim J.P. (2011). The synthesis of thermally-stable red dyes for LCD color filters and analysis of their aggregation and spectral properties. Dyes Pigment..

[27] Li X., Sinks L.E., Rybtchinski B., Wasielewski M.R. (2004). Ultrafast aggregate-to-aggregate energy transfer within self-assembled light-harvesting columns of zinc phthalocyanine tetrakis (perylenediimide). J. Am. Chem. Soc..

[28] Rybtchinski B., Sinks L.E., Wasielewski M.R. (2004). Combining light-harvesting and charge separation in a self-assembled artificial photosynthetic system based on perylenediimide chromophores. J. Am. Chem. Soc..

[29] Kozma E., Kotowski D., Catellani M., Luzzati S., Famulari A., Bertini F. (2013). Synthesis and characterization of new electron acceptor perylene diimide molecules for photovoltaic applications. Dyes Pigment..

[30] Dinçalp H., Aşkar Z., Zafer C., İçli S. (2011). Effect of side chain substituents on the electron injection abilities of unsymmetrical perylene diimide dyes. Dyes Pigment..

[31] Ramanan C., Semigh A.L., Anthony J.E., Marks T.J., Wasielewski M.R. (2012). Competition between singlet fission and charge separation in solution-processed blend films of 6,13-bis (triisopropylsilylethynyl)-pentacene with sterically-encumbered perylene-3,4:9,10-bis (dicarboximide)s. J. Am. Chem. Soc..

[32] Kozma E., Catellani M. (2013). Perylene diimides based materials for organic solar cells. Dyes Pigment..

[33] Kaur B., Bhattacharya S.N., Henry D.J. (2013). Interpreting the near-infrared reflectance of a series of perylene pigments. Dyes Pigment..

[34] Würthner F. (2004). Perylene bisimide dyes as versatile building blocks for functional supramolecular architectures. Chem. Commun..

[35] Chang C.W., Tsai H.Y., Chen K.Y. (2014). 1,6-Dinitroperylene bisimide dyes: synthesis, characterization and photophysical properties. J. Chin. Chem. Soc..

[36] Chen K.Y., Chang C.W. (2014). 1,7-Bis-(*N*,*N*-dialkylamino)perylene bisimides: Facile synthesis and characterization as near-infrared fluorescent dyes. Materials.

[37] Tsai H.-Y., Chang C.-W., Chen K.-Y. (2014). 1,6- And 1,7-regioisomers of dinitro- and diamino-substituted perylene bisimides: Synthesis, photophysical and electrochemical properties. Tetrahedron Lett..

[38] Chang C.W., Tsai H.Y., Chen K.Y. (2014). Green perylene bisimide dyes: synthesis, photophysical and electrochemical properties. Materials.

[39] Chen K.Y., Chang C.W. (2014). Highly soluble monoamino-substituted perylene tetracarboxylic dianhydrides: synthesis, optical and electrochemical properties. Int. J. Mol. Sci..

[40] Rajasingh P., Cohen R., Shirman E., Shimon L.J.W., Rybtchinski B. (2007). Selective bromination of perylene diimides under mild conditions. J. Org. Chem..

[41] Chen K.Y., Fang T.C., Chang M.J. (2011). Synthesis, photophysical and electrochemical properties of 1-aminoperylene bisimides. Dyes Pigment..

[42] Tsai H.Y., Chen K.Y. (2013). 1,7-Diaminoperylene bisimides: Synthesis, optical and electrochemical properties. Dyes Pigment..

[43] Ahrens M.J., Tauber M.J., Wasielewski M.R. (2006). Bis(*n*-octylamino)perylene-3,4:9,10-bis(dicarboximide)s and their radical cations: Synthesis, electrochemistry, and ENDOR spectroscopy. J. Org. Chem..

[44] Zhao C., Zhang Y., Li R., Li X., Jiang J. (2007). Di(alkoxy)- and di(alkylthio)-substituted perylene-3,4;9,10-tetracarboxy diimides with tunable electrochemical and photophysical properties. J. Org. Chem..

[45] Zhang X., Pang S., Zhang Z., Ding X., Zhang S., He S., Zhan C. (2012). Facile synthesis of 1-bromo-7-alkoxyl perylene diimide dyes: Toward unsymmetrical functionalizations at the 1,7-positions. Tetrahedron Lett..

[46] Dhokale B., Gautam P., Misra R. (2012). Donor-acceptor perylenediimide-ferrocene conjugates: Synthesis, photophysical, and electrochemical properties. Tetrahedron Lett..

[47] Miasojedovasa A., Kazlauskasa K., Armonaitea G., Sivamuruganb V., Valiyaveettilb S., Grazuleviciusc J.V., Jursenasa S. (2012). Concentration effects on emission of bay-substituted perylene diimide derivatives in a polymer matrix. Dyes Pigment..

[48] Chen K.Y., Chow T.J. (2010). 1,7-Dinitroperylene bisimides: Facile synthesis and characterization as *n*-type organic semiconductors. Tetrahedron Lett..

[49] Chen Z.J., Wang L.M., Zou G., Zhang L., Zhang G.J., Cai X.F., Teng M.S. (2012). Colorimetric and ratiometric luorescent chemosensor for fluoride ion based on perylene diimide derivatives. Dyes Pigment..

[50] Kong X., Gao J., Ma T., Wang M., Zhang A., Shi Z., Wei Y. (2012). Facile synthesis and replacement reactions of mono-substituteded perylene bisimide dyes. Dyes Pigment..

[51] Dubey R.K., Efimov A., Lemmetyinen H. (2011). 1,7- and 1,6-regioisomers of diphenoxy and dipyrrolidinyl substituted perylene diimides: Synthesis, separation, characterization, and comparison of electrochemical and optical properties. Chem. Mater..

[52] Würthner F., Stepanenko V., Chen Z., Saha-Möller C.R., Kocher N., Stalke D. (2004). Preparation and characterization of regioisomerically pure 1,7-disubstituted perylene bisimide dyes. J. Org. Chem..

[53] Dubey R.K., Niemi M., Kaunisto K., Efimov A., Tkachenko N.V., Lemmetyinen H. (2013). Direct evidence of significantly different chemical behavior and excited-state dynamics of 1,7- and 1,6-regioisomers of pyrrolidinyl-substituted perylene diimide. Chem. Eur. J..

[54] Sengupta S., Dubey R.K., Hoek R.W.M., van Eeden S.P.P., Gunbaş D.D., Grozema F.C., Sudhölter E.J.R., Jager W.F. (2014). Synthesis of regioisomerically pure 1,7-dibromoperylene-3,4,9,10-tetracarboxylic acid derivatives. J. Org. Chem..

[55] Lakowicz J.R. (1999). Principles of Fluorescence Spectroscopy.

