# Supplementary material for: 1,6- and 1,7-Regioisomers of Highly Soluble Amino-Substituted Perylene Tetracarboxylic Dianhydrides: Synthesis, Optical and Electrochemical Properties
Source: Materials (Basel). 2015 Aug 3;8(8):4943–60. doi: 10.3390/ma8084943 (PMC5455523; doi:10.3390/ma8084943)
Supplement: Supplementary file 1 [file materials-08-04943-s001.pdf]

Supplementary Materials

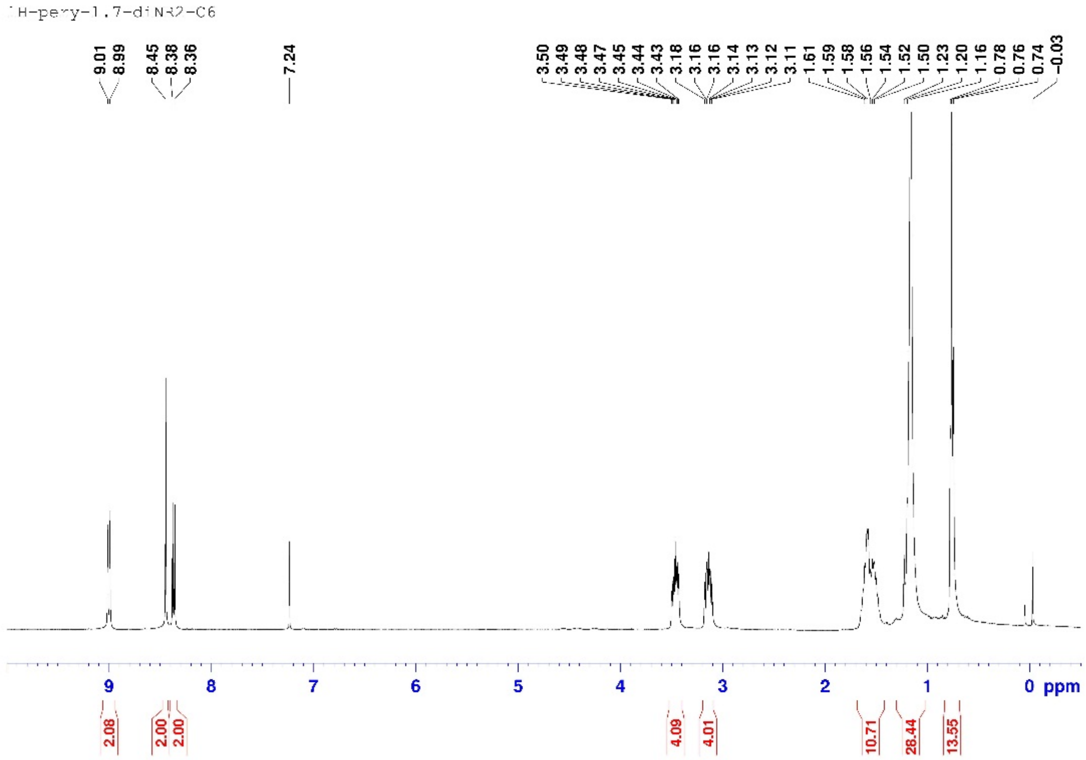

Figure S1. ^{1}H NMR of **1a**.

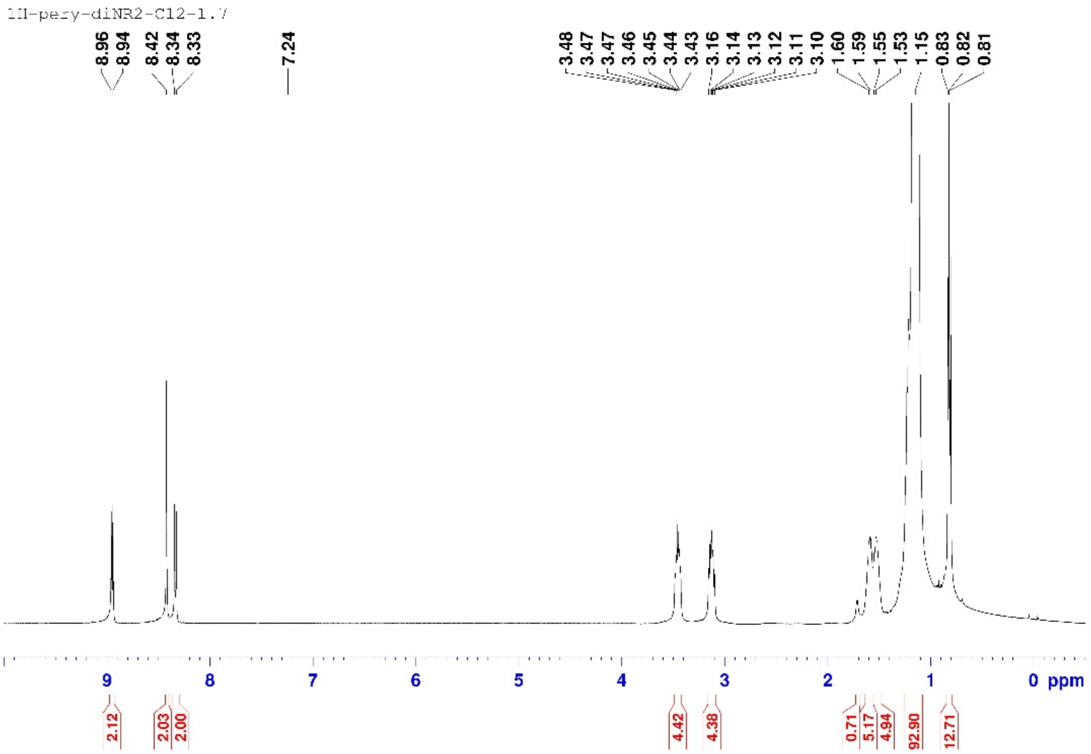

Figure S2. ^{1}H NMR of **1b**.

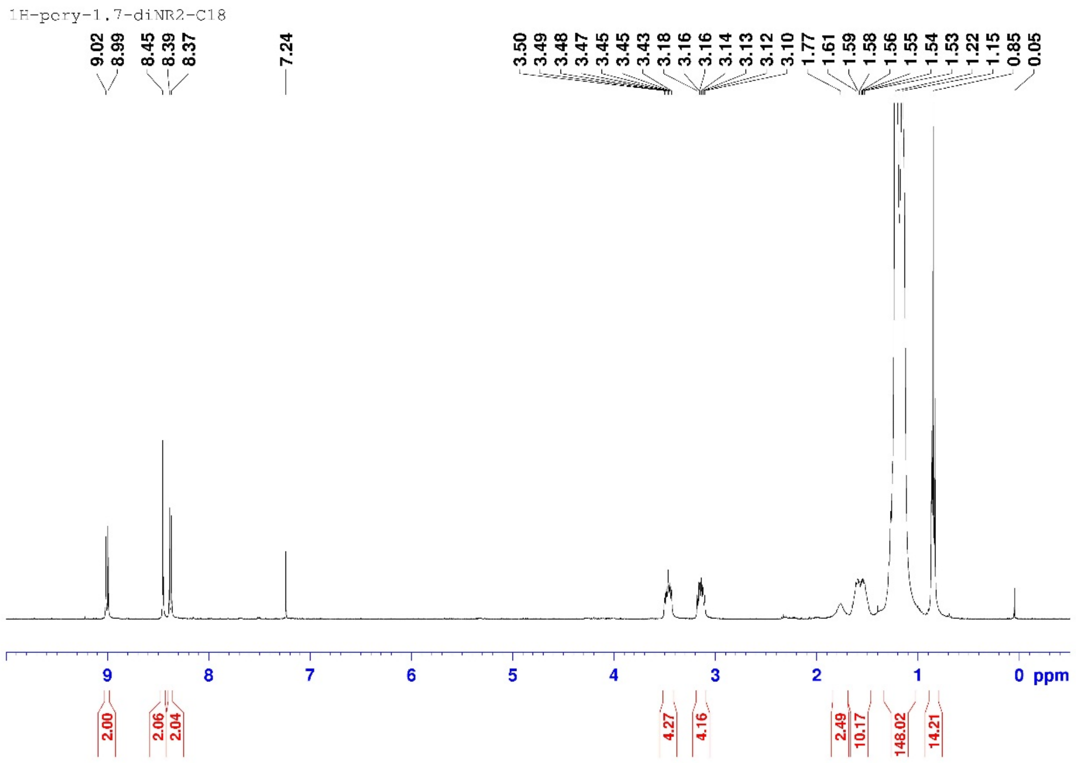

Figure S3. ^{1}H NMR of **1c**.

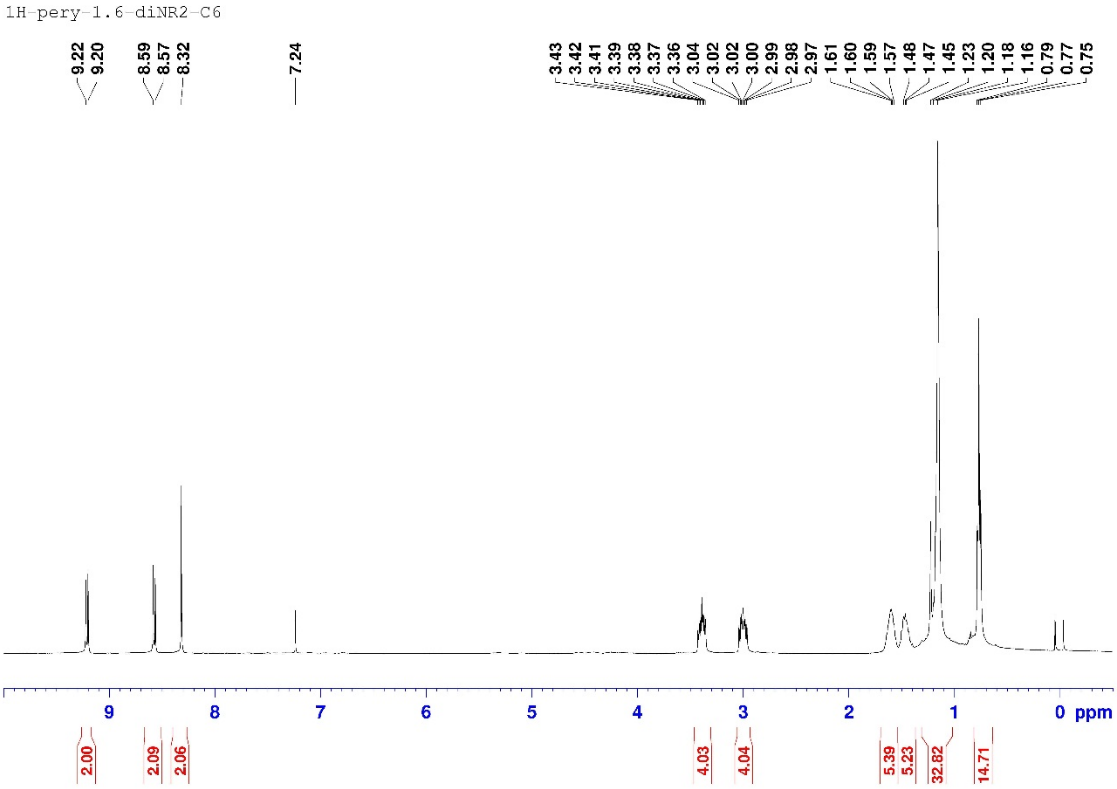

Figure S4. ^{1}H NMR of **2a**.

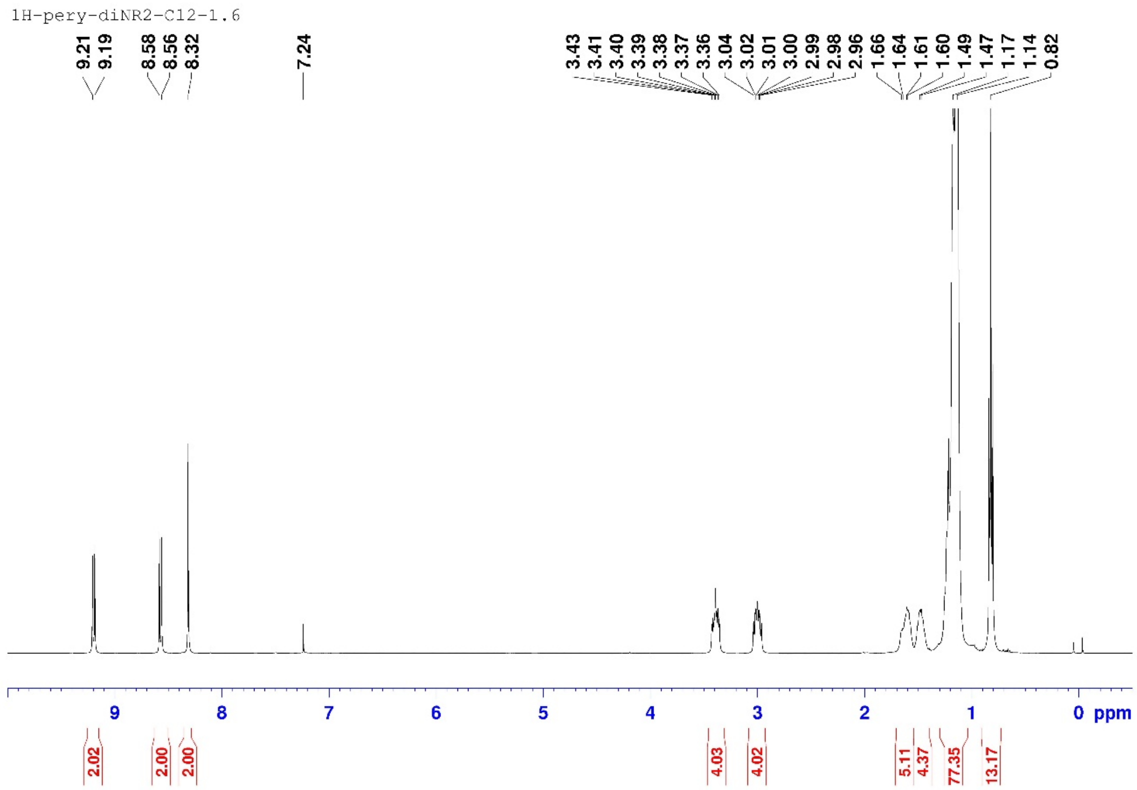

Figure S5. ^{1}H NMR of **2b**.

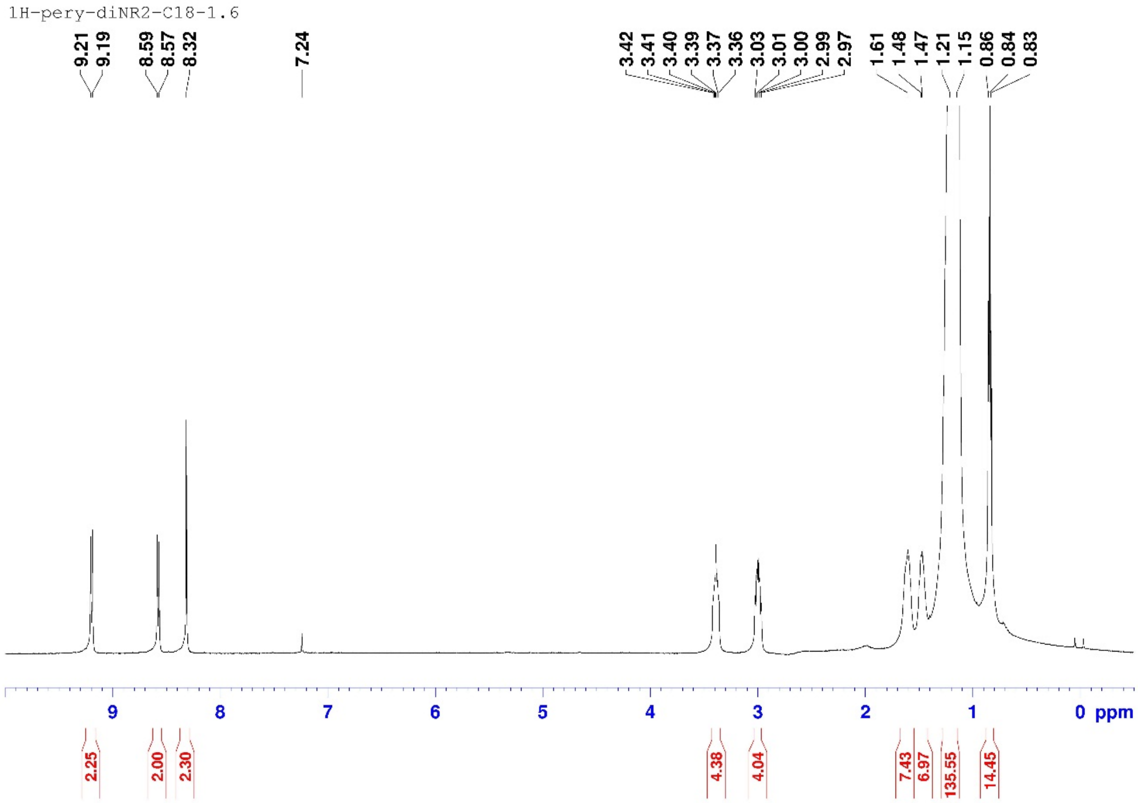

Figure S6. ^{1}H NMR of **2c**.

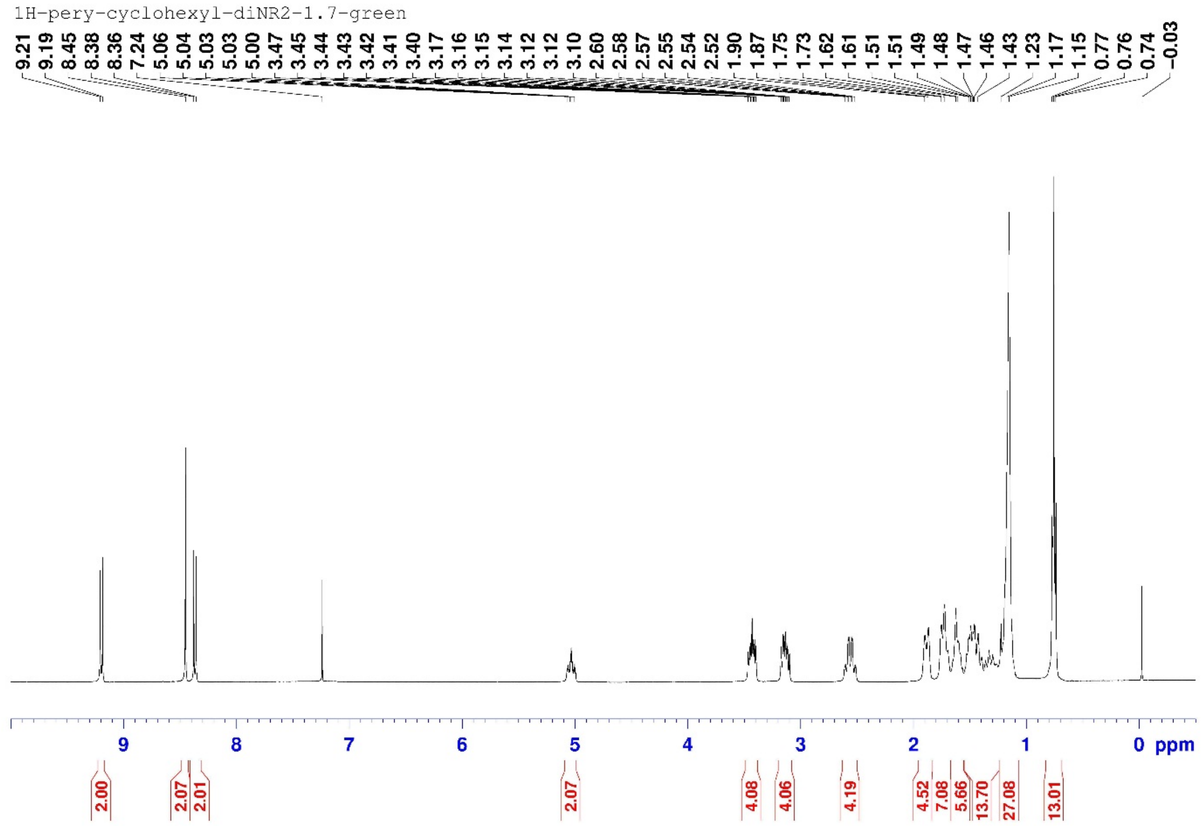

Figure S7. ^{1}H NMR of **3a**.

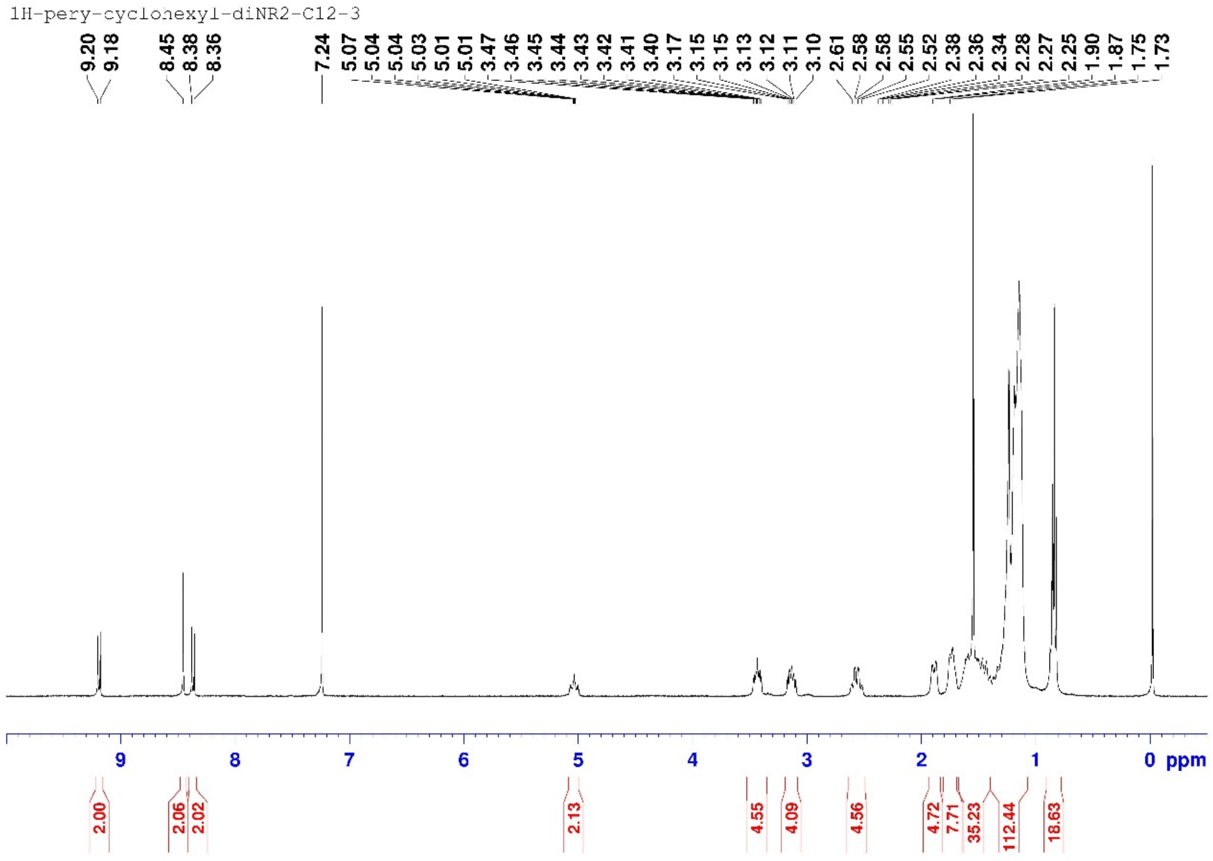

Figure S8. ^{1}H NMR of **3b**.

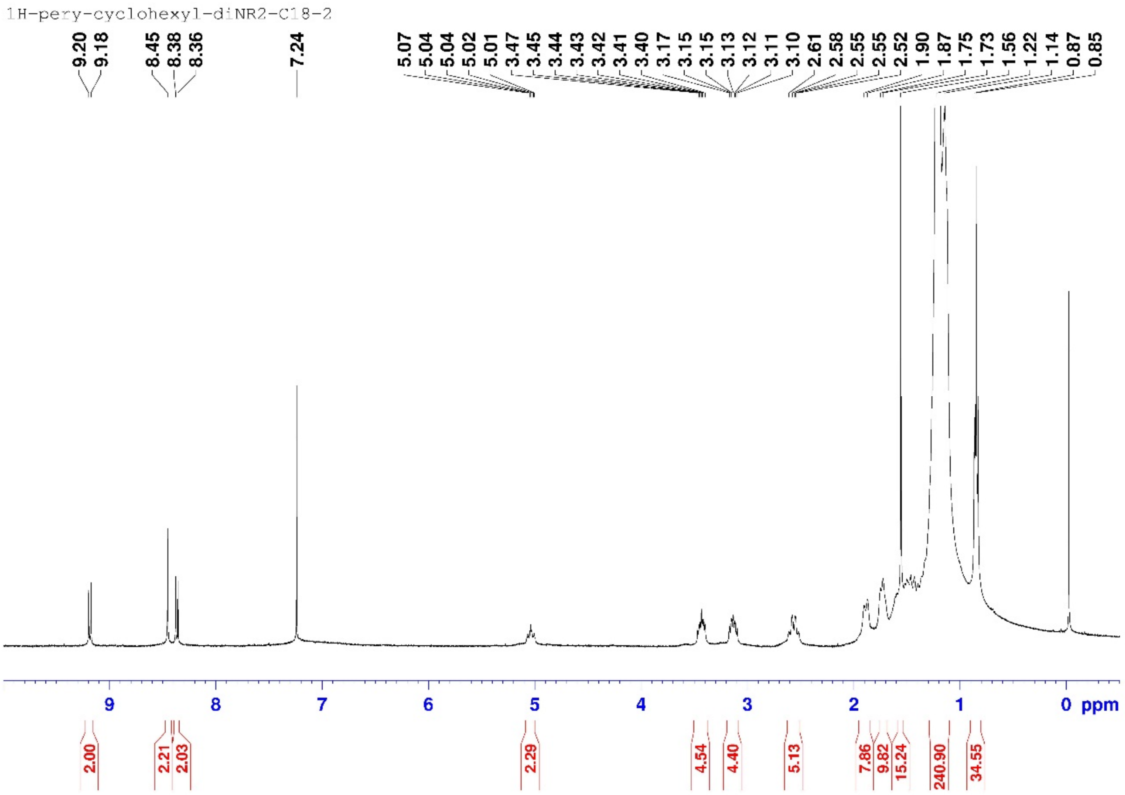

Figure S9. ^{1}H NMR of **3c**.

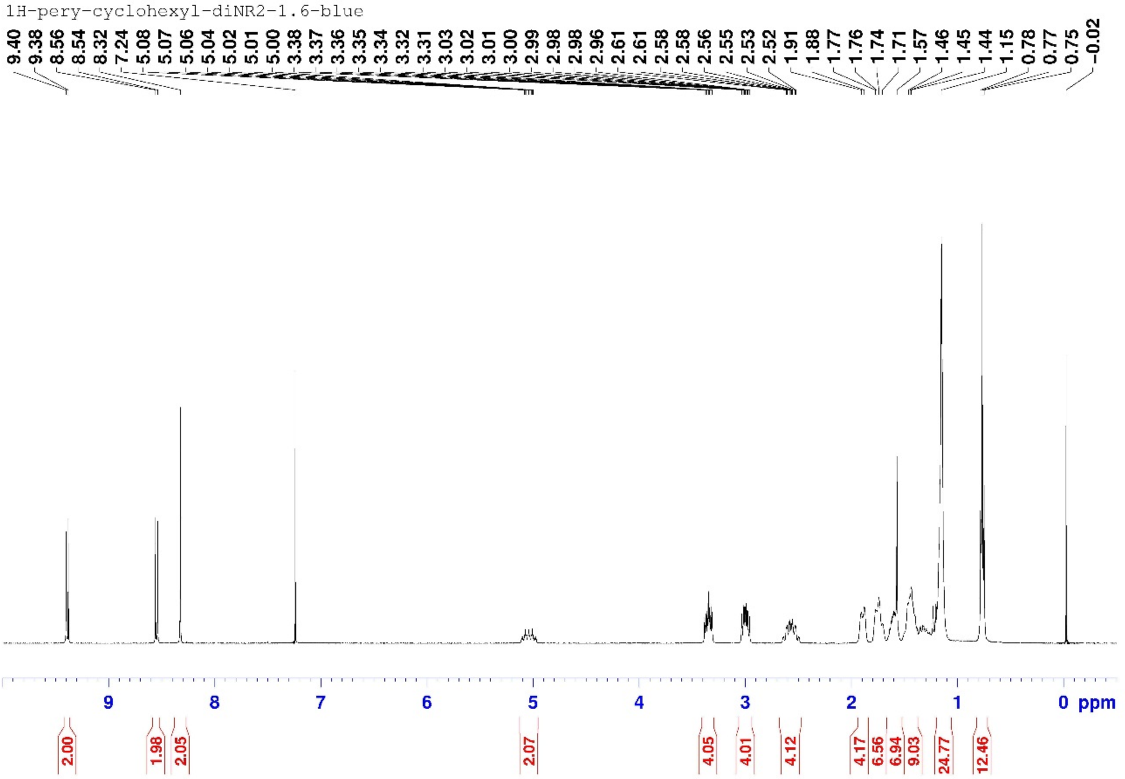

Figure S10. ^{1}H NMR of **4a**.

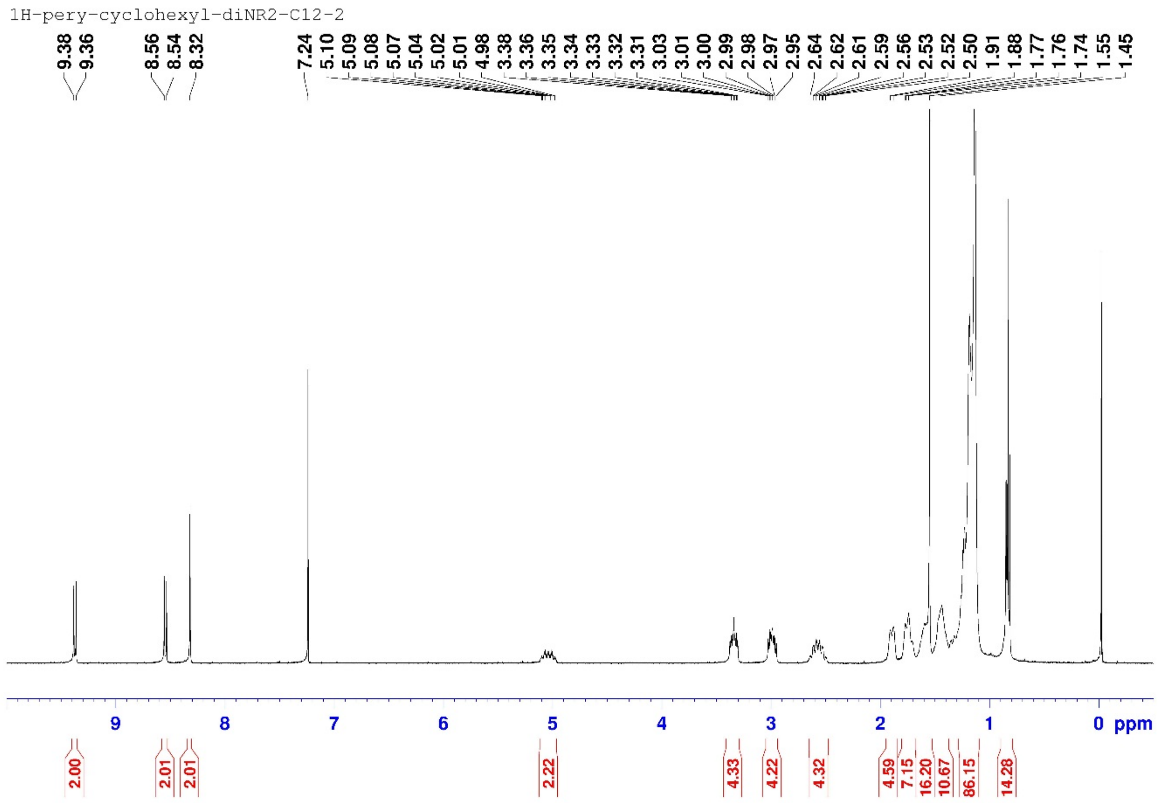

Figure S11. ^{1}H NMR of **4b**.

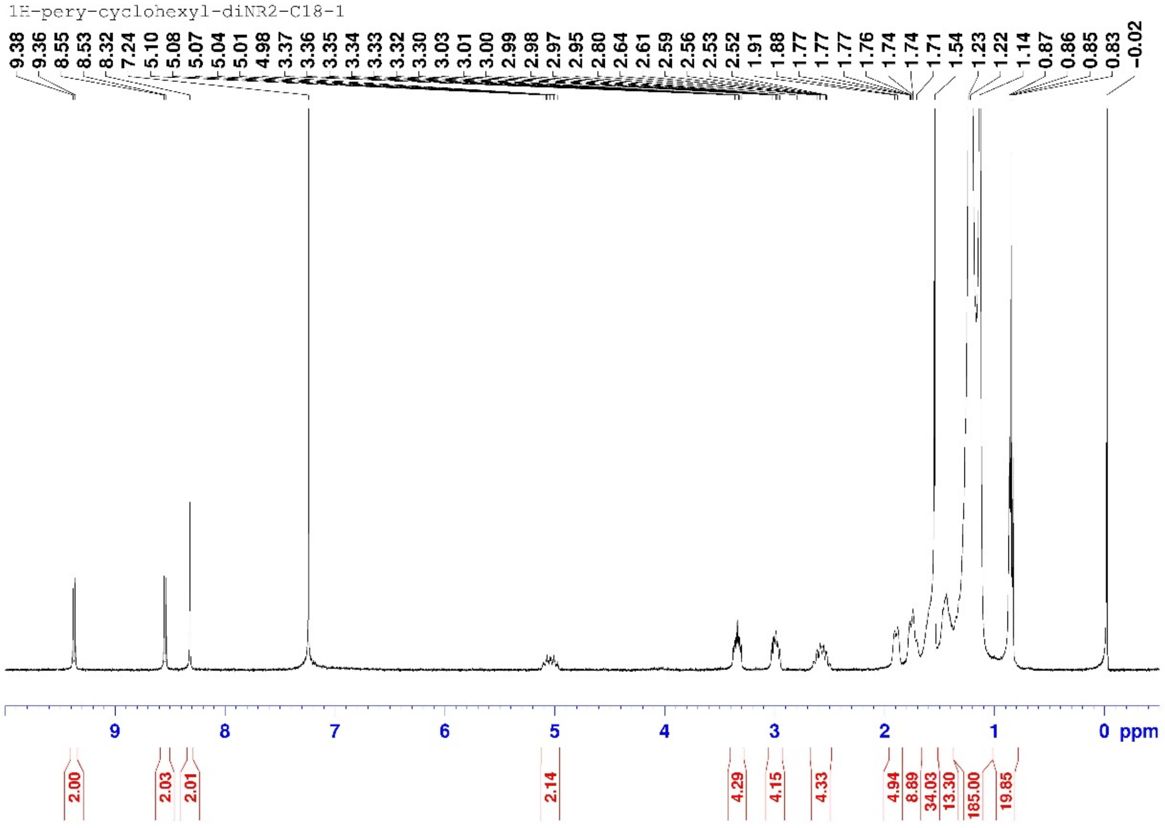

Figure S12. ^{1}H NMR of **4c**.

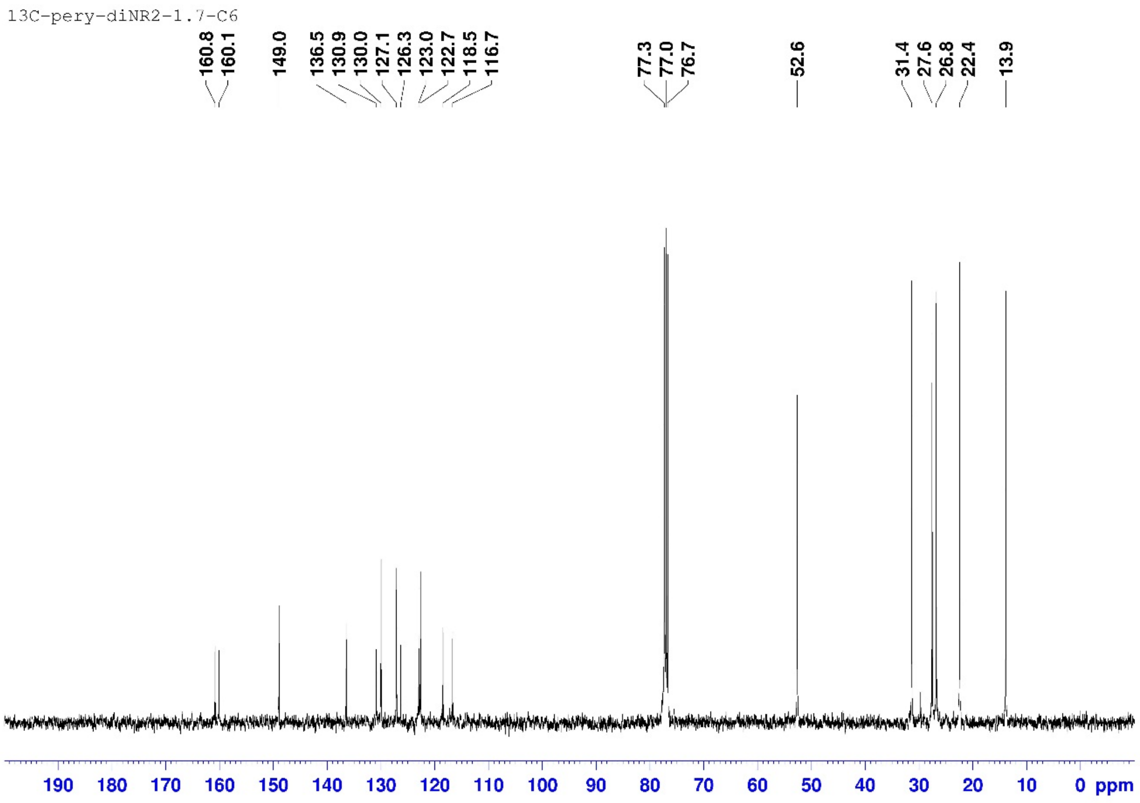

Figure S13. ^{13}C NMR of **1a**.

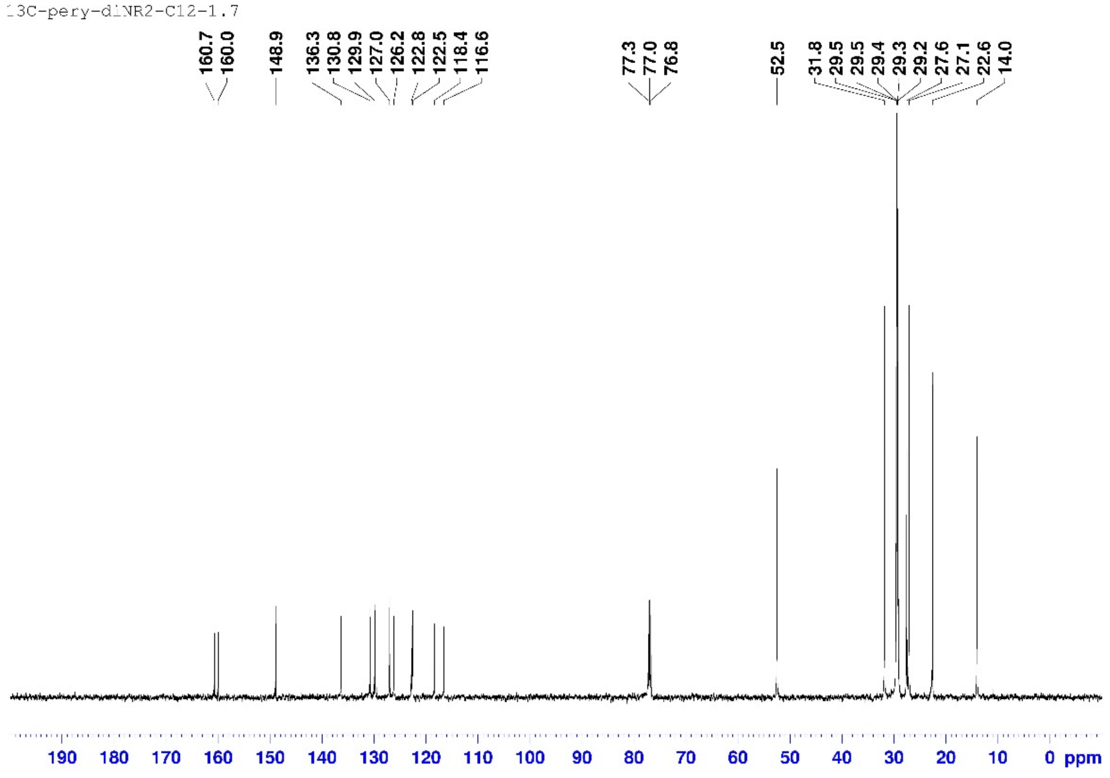

Figure S14. ^{13}C NMR of **1b**.

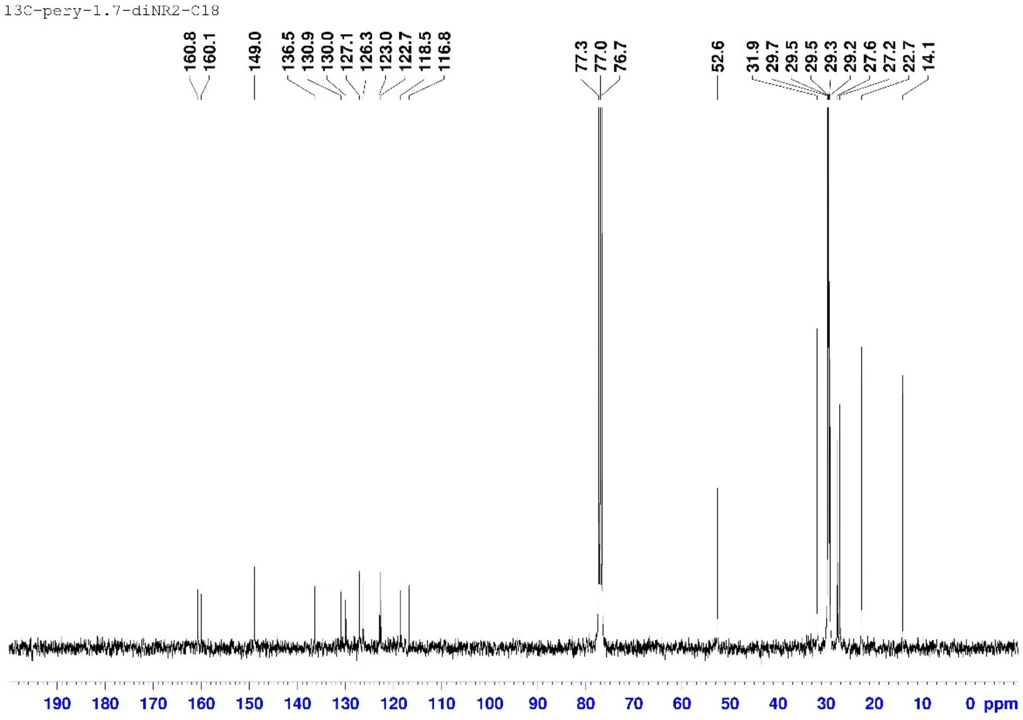

Figure S15. ^{13}C NMR of **1c**.

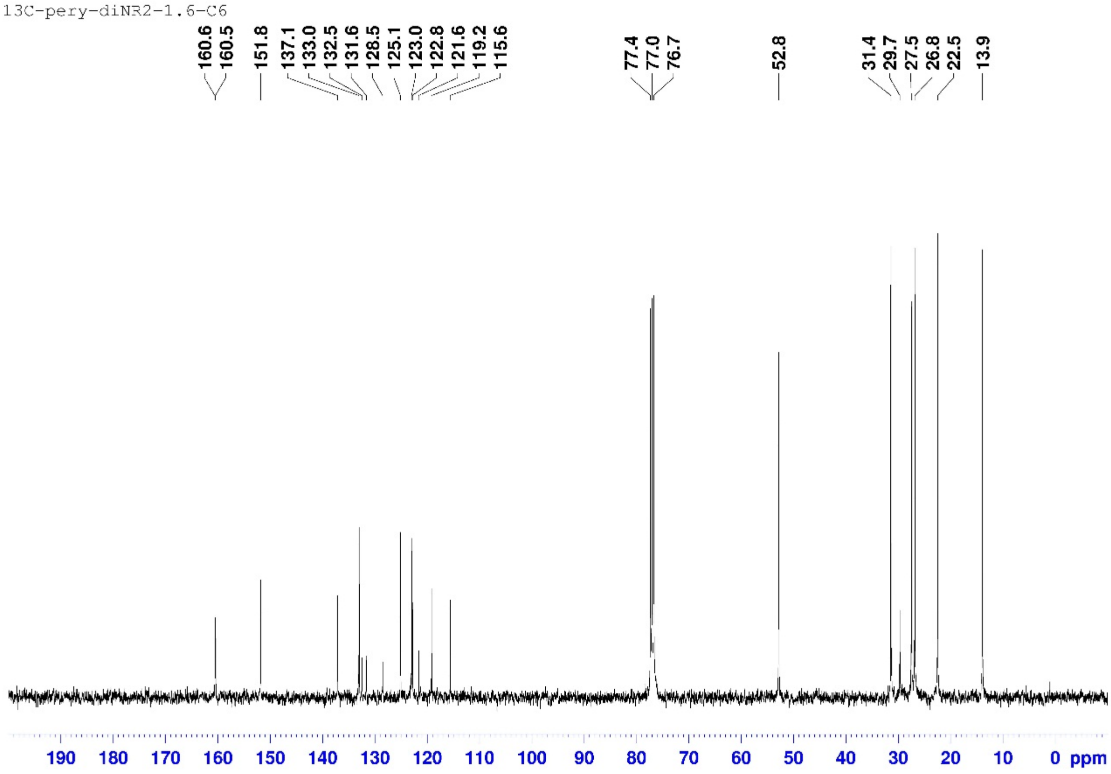

Figure S16. ^{13}C NMR of **2a**.

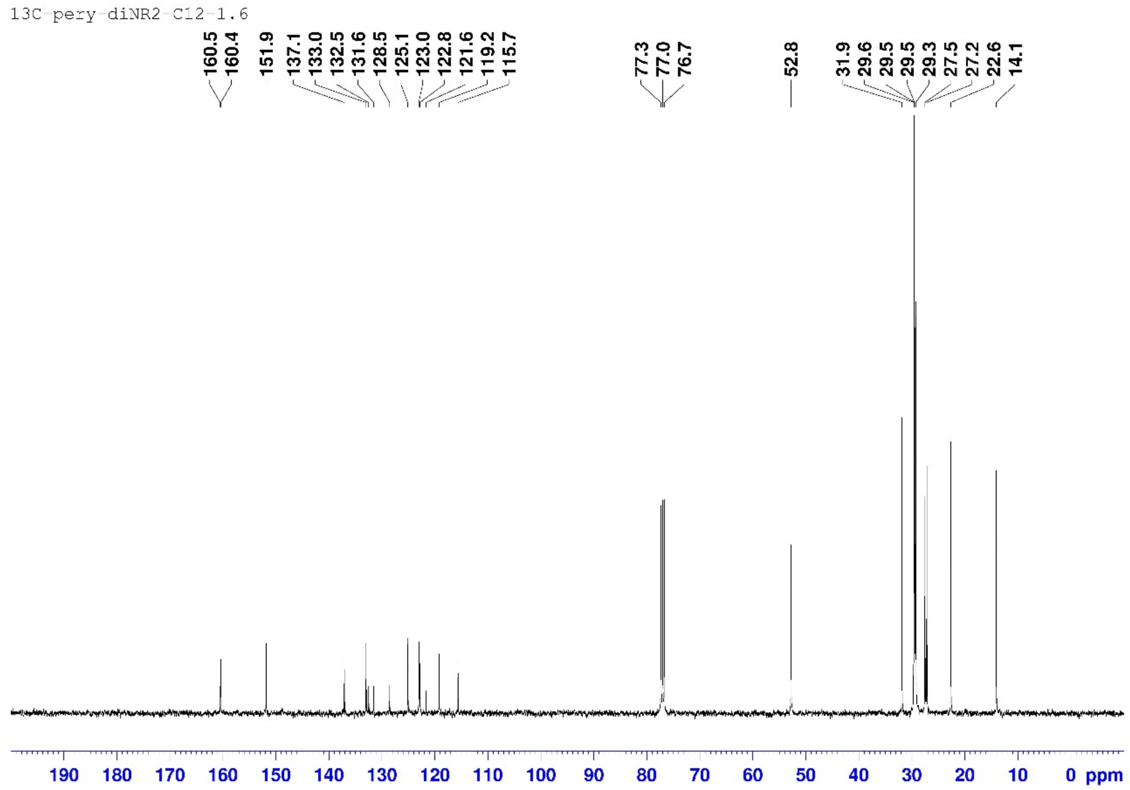

Figure S17. ^{13}C NMR of **2b**.

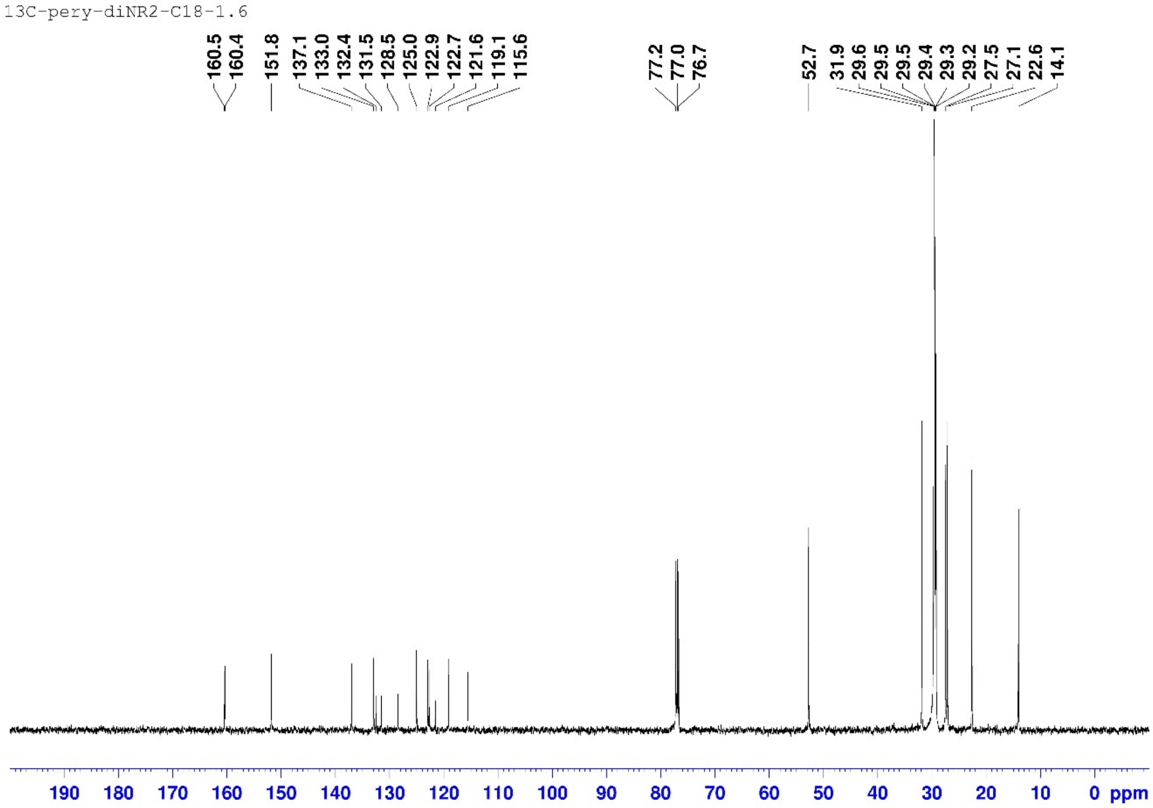

Figure S18. ^{13}C NMR of **2c**.

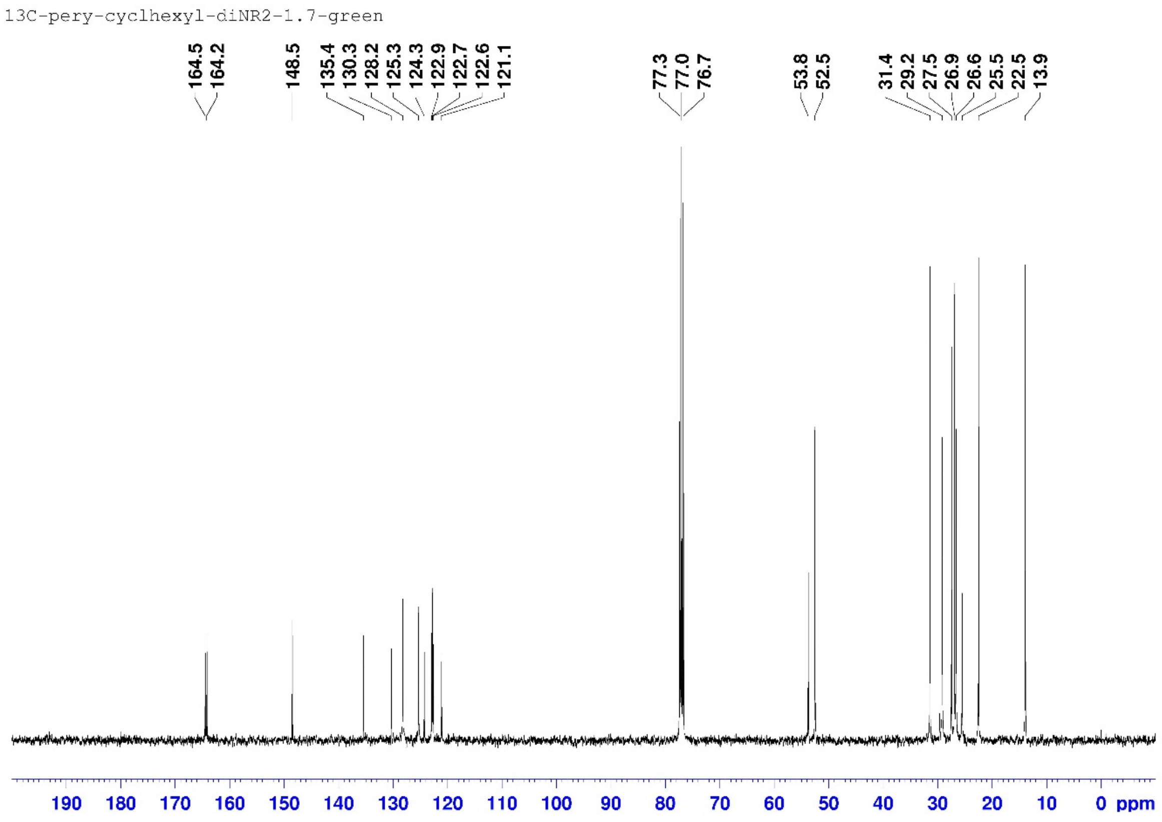

Figure S19. ^{13}C NMR of **3a**.

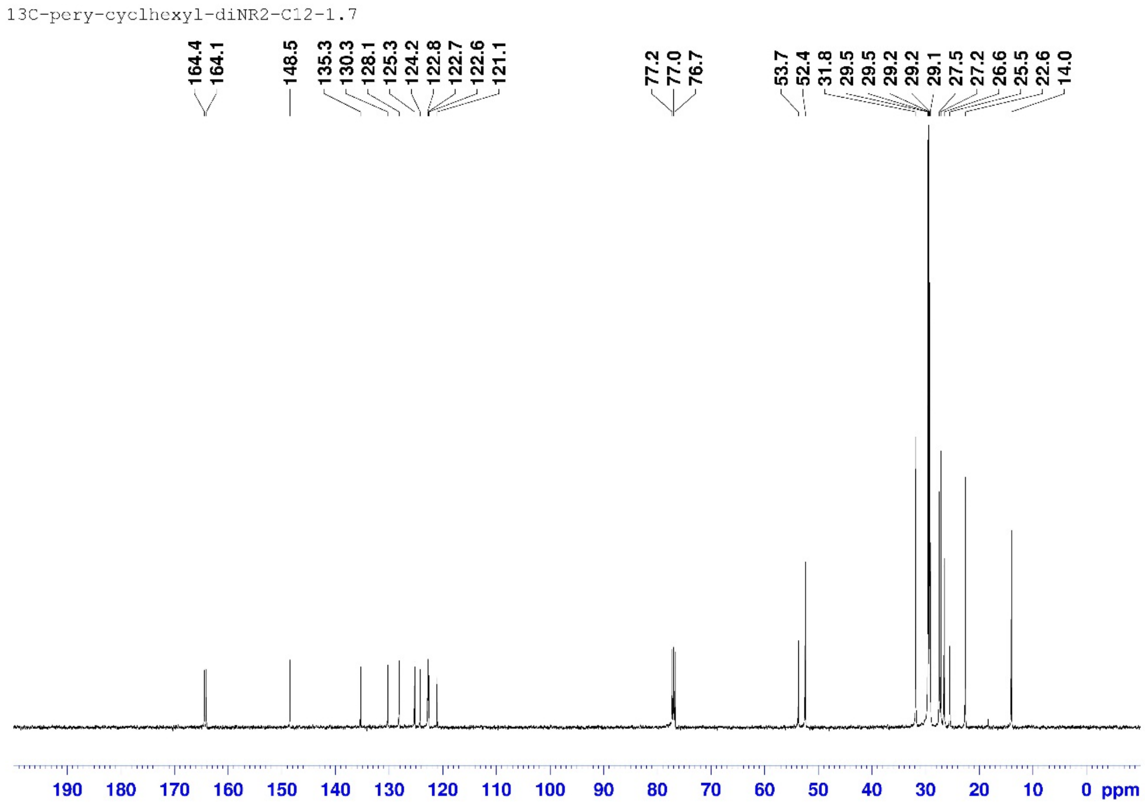

Figure S20. ^{13}C NMR of **3b**.

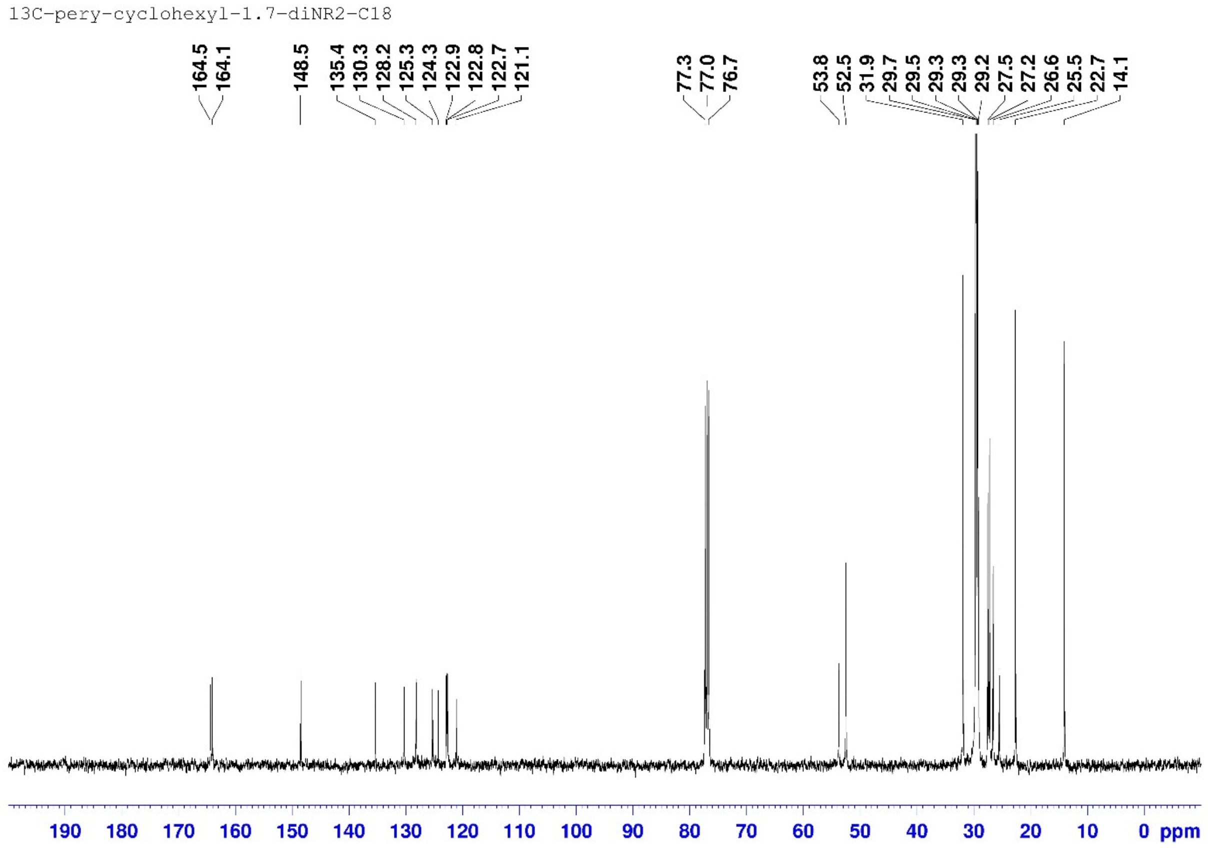

Figure S21. ^{13}C NMR of **3c**.

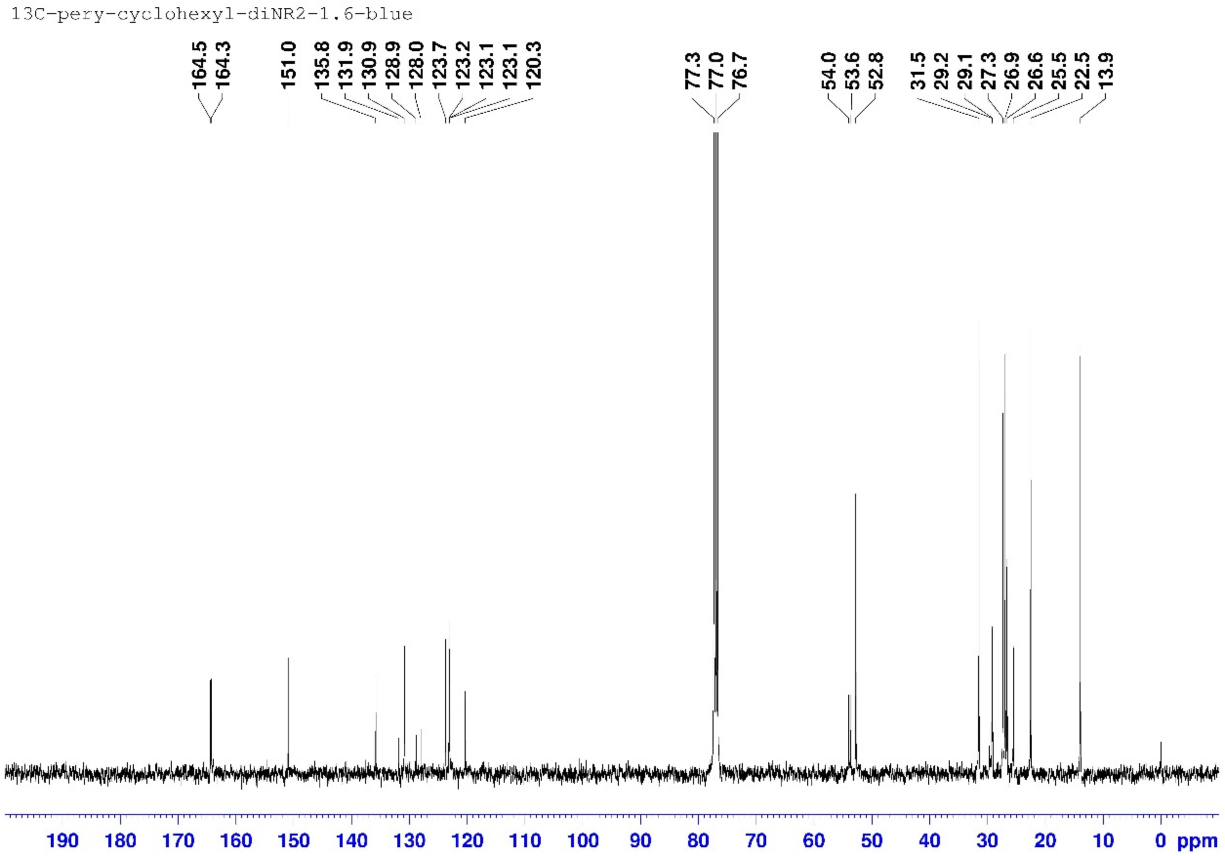

Figure S22. ^{13}C NMR of **4a**.

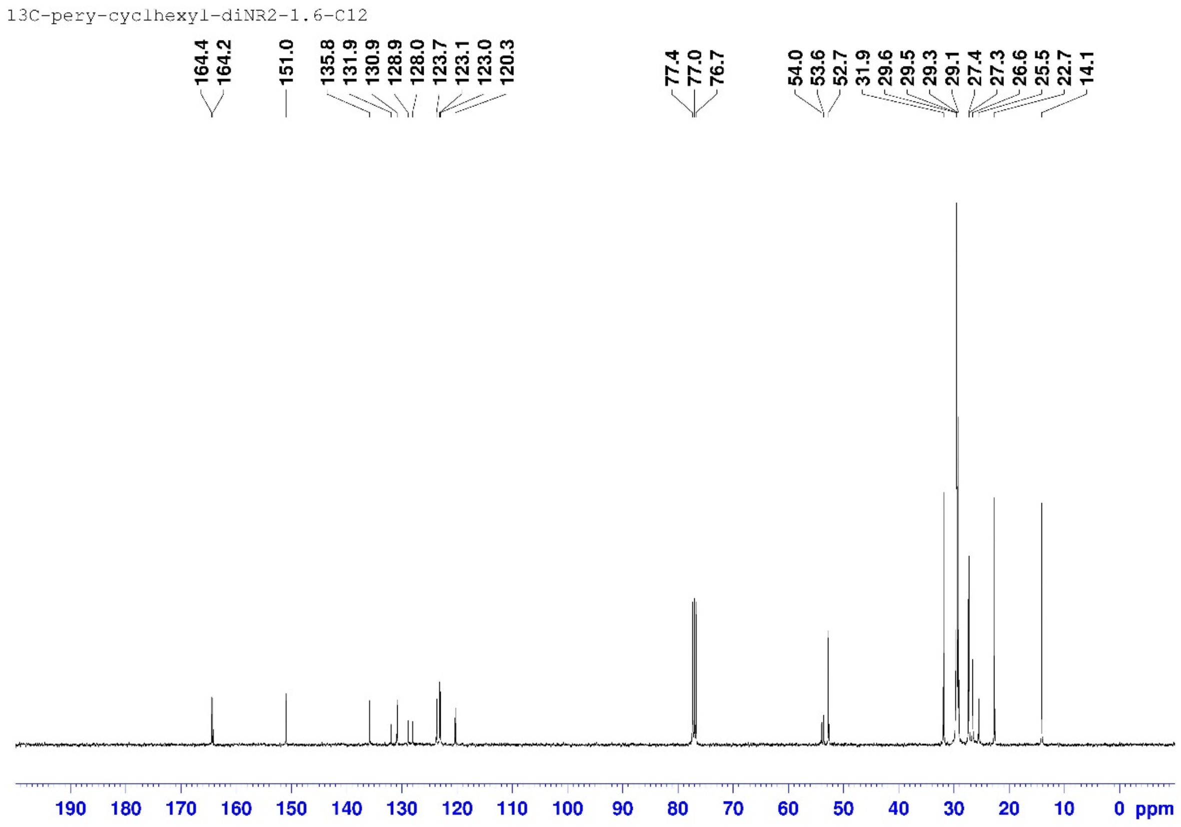

Figure S23. ^{13}C NMR of **4b**.

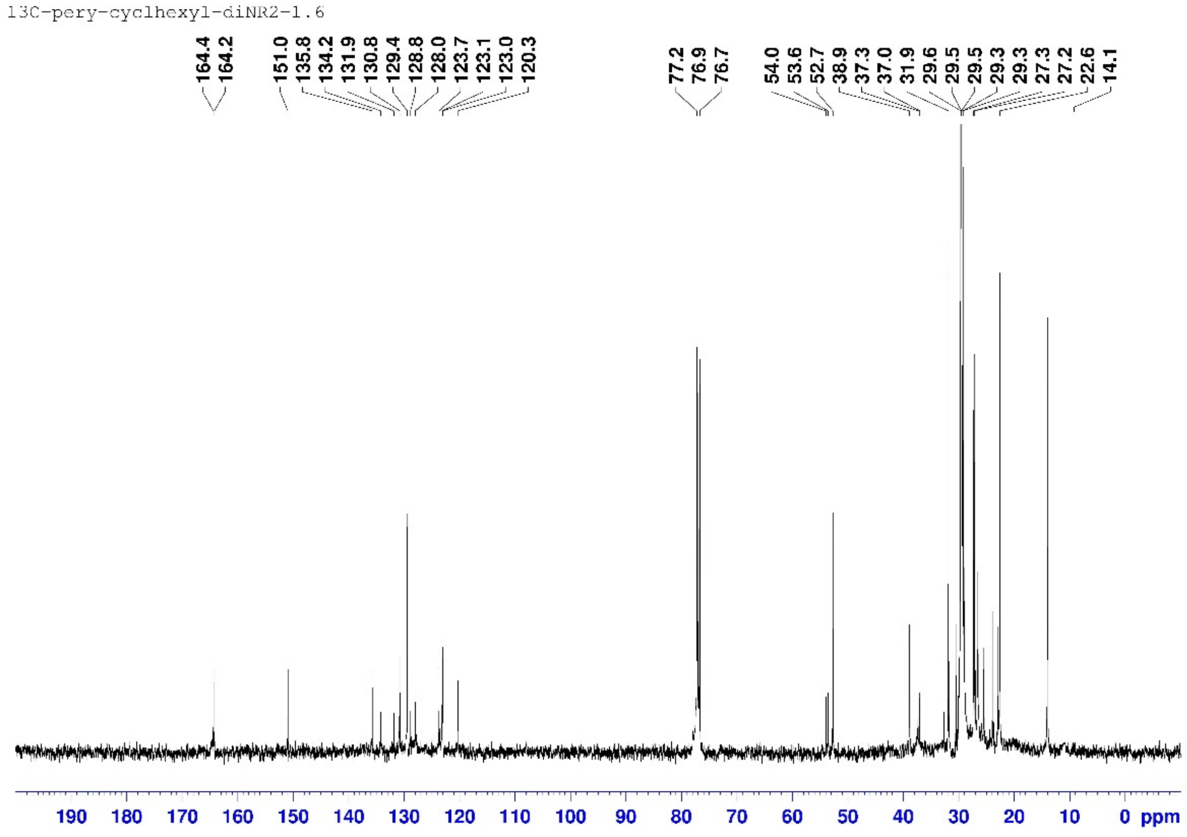

Figure S24. ^{13}C NMR of **4c**.

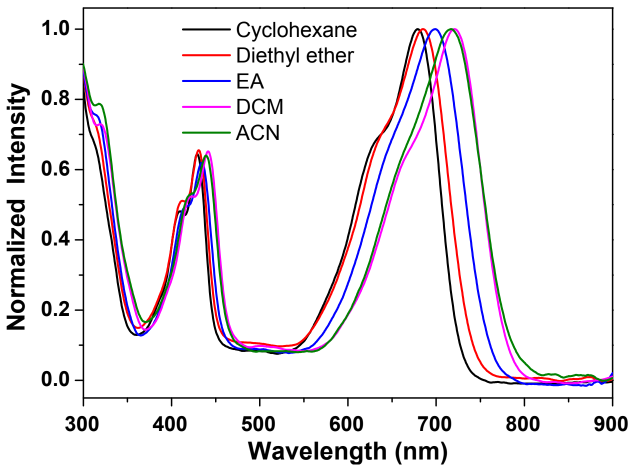

Figure S25. Normalized absorption spectra of **1b** in various solvents.

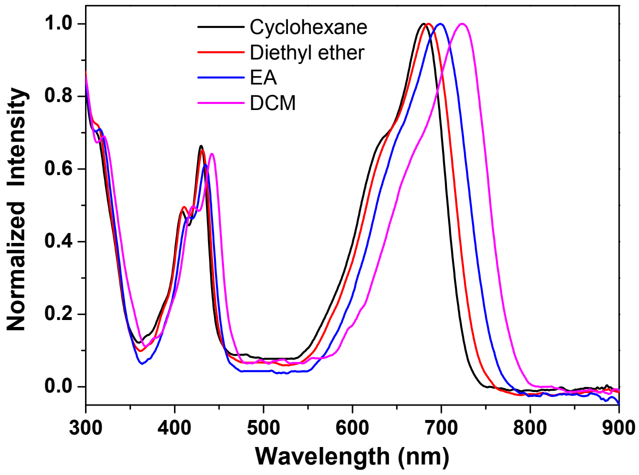

Figure S26. Normalized absorption spectra of **1c** in various solvents.

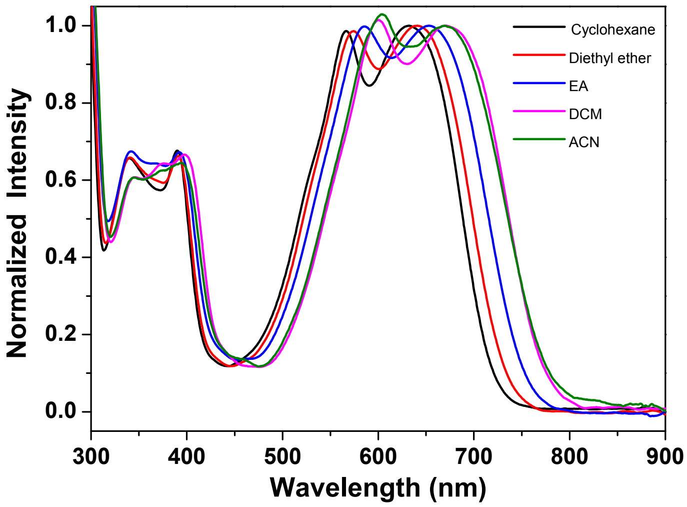

Figure S27. Normalized absorption spectra of **2b** in various solvents.

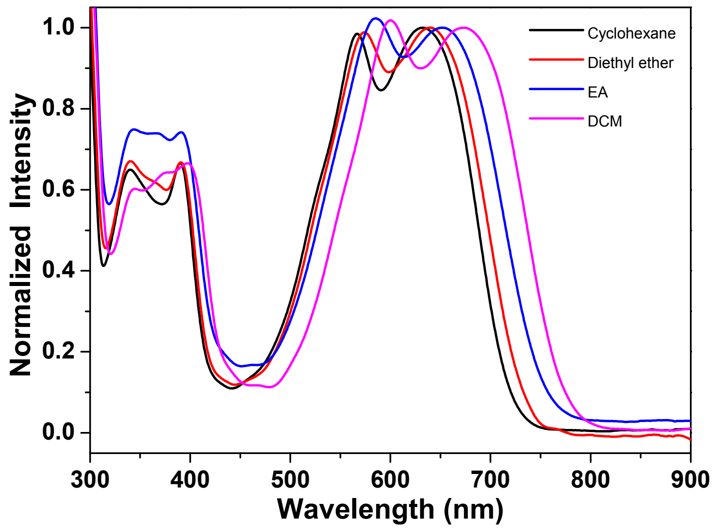

Figure S28. Normalized absorption spectra of **2c** in various solvents.

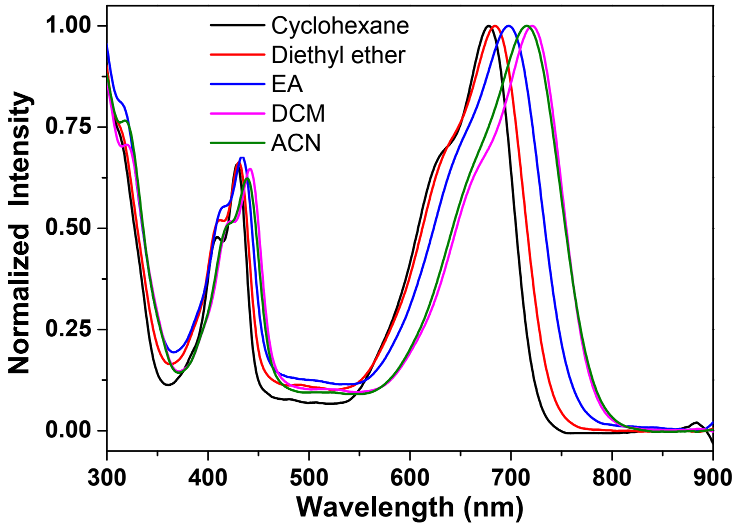

Figure S29. Normalized absorption spectra of **1a** in various solvents.

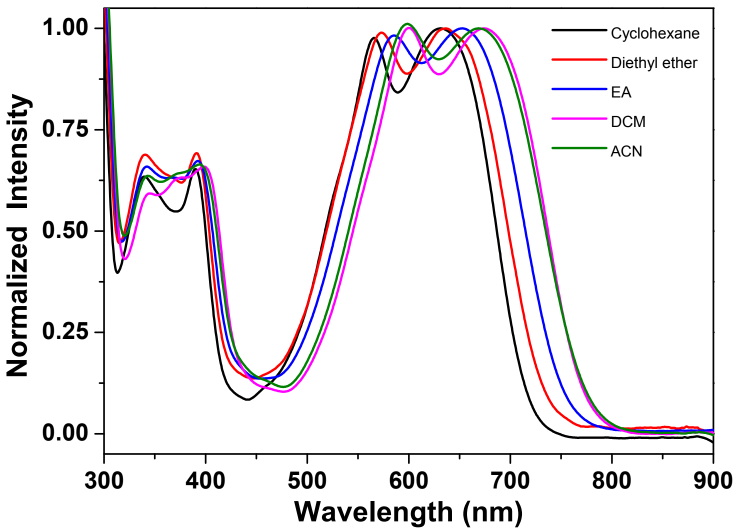

Figure S30. Normalized absorption spectra of **2a** in various solvents.

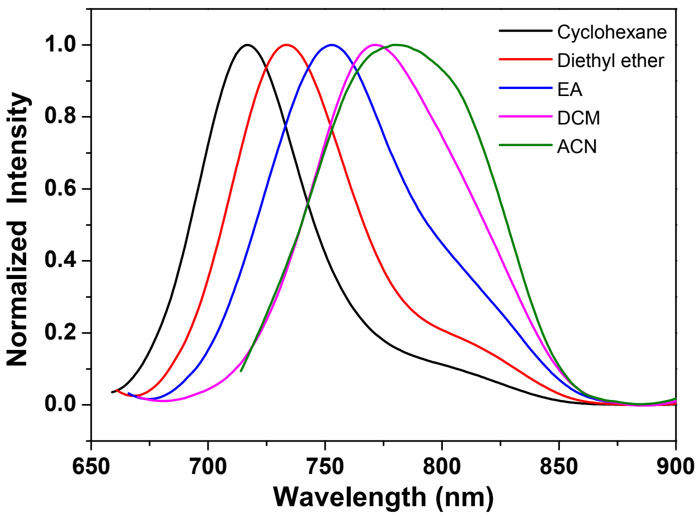

Figure S31. Normalized emission spectra of **1b** in various solvents.

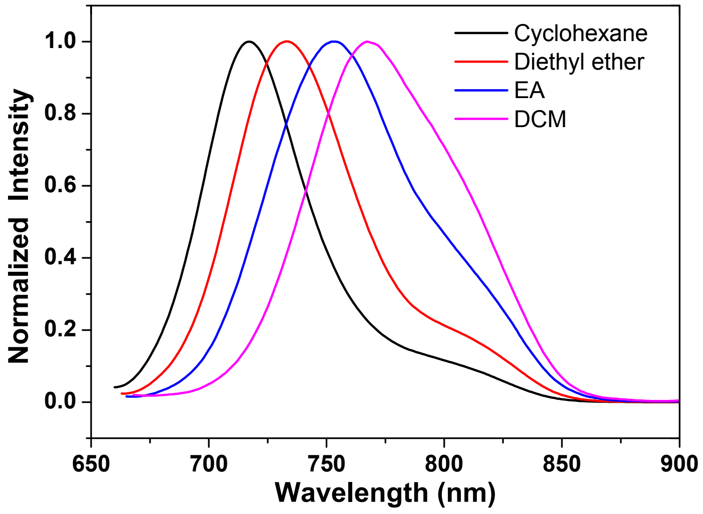

Figure S32. Normalized emission spectra of **1c** in various solvents.

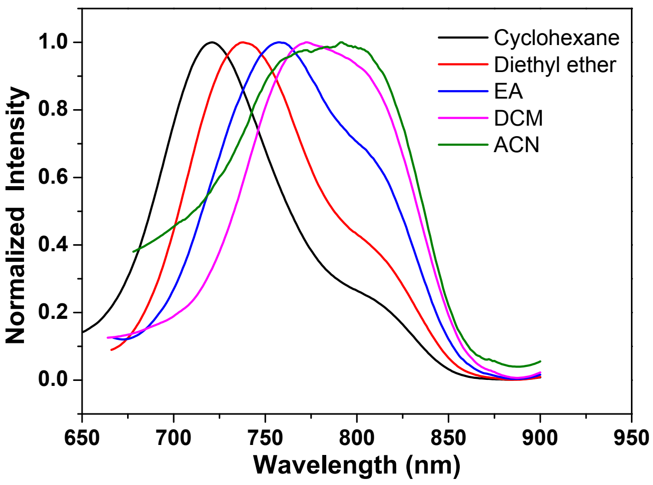

Figure S33. Normalized emission spectra of **2b** in various solvents.

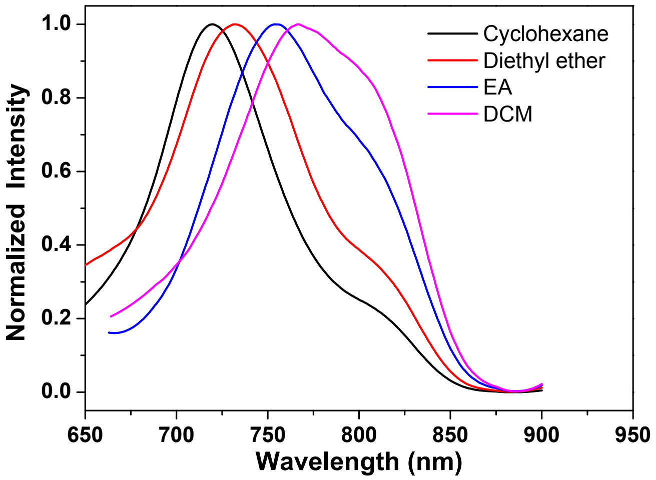

Figure S34. Normalized emission spectra of **2c** in various solvents.

Table S1. Summary of optical absorption and emission properties of **3a**–**3c** in various solvents.

3a/3b/3c	λ_{abs} (nm) [a]	λ_{em} (nm) [a]	Stokes shift (nm)	Φ [b] × 10^2
cyclohexane	667/670/670	711/714/716	44/44/48	3.03/4.74/3.14
diethyl ether	675/676/676	726/725/726	51/46/51	0.44/0.80/0.92
ethyl acetate	687/688/687	741/740/740	57/54/55	0.22/0.41/0.42
dichloromethane	698/702/701	755/758/758	52/55/54	0.20/0.40/0.41
acetonitrile	699/703/703	760/760/761	61/56/57	0.25/0.26/0.26

[a] Measured at 2 × 10^{-5} M; [b] Determined with *N*,*N*'-dioctyl-3,4,9,10-perylenedicarboximide as reference [42].

Table S2. Summary of optical absorption and emission properties of **4a**–**4c** in various solvents.

4a/4b/4c	λ_{abs} (nm) [a]	λ_{em} (nm) [a]	Stokes shift (nm)	Φ [b] × 10^3
cyclohexane	625/624/620	721/720/712	96/96/92	2.42/3.72/5.79
diethyl ether	632/633/631	736/733/734	104/100/103	0.61/0.82/0.98
ethyl acetate	638/639/640	747/748/747	109/109/107	0.47/0.63/0.58
dichloromethane	658/655/658	791/788/789	133/133/131	0.33/0.45/0.40
acetonitrile	656/658/657	800/796/798	144/138/141	0.17/0.36/0.34

[a] Measured at 2 × 10^{-5} M; [b] Determined with *N*,*N*'-dioctyl-3,4,9,10-perylenedicarboximide as reference [42].
